# Circular RNAs in hepatocellular carcinoma: biogenesis, function, and pathology

**DOI:** 10.3389/fgene.2023.1106665

**Published:** 2023-07-07

**Authors:** Guocheng Rao, Xi Peng, Yan Tian, Xianghui Fu, Yuwei Zhang

**Affiliations:** ^1^ Department of Endocrinology and Metabolism, Cancer Center West China Hospital, Sichuan University, Chengdu, Sichuan, China; ^2^ Department of Endocrinology and Metabolism, West China Hospital, West China Medical School, Sichuan University, Chengdu, Sichuan, China

**Keywords:** HCC, circRNA, miRNA sponge, tumor microenvironment, biomarker, drug resistance

## Abstract

Hepatocellular carcinoma (HCC) is one of the most common causes of cancer-related death worldwide. Both genetic and environmental factors through a multitude of underlying molecular mechanisms participate in the pathogenesis of HCC. Recently, numerous studies have shown that circular RNAs (circRNAs), an emerging class of non-coding RNAs characterized by the presence of covalent bonds linking 3’ and 5’ ends, play an important role in the initiation and progression of cancers, including HCC. In this review, we outline the current status of the field of circRNAs, with an emphasis on the functions and mechanisms of circRNAs in HCC and its microenvironment. We also summarize and discuss recent advances of circRNAs as biomarkers and therapeutic targets. These efforts are anticipated to throw new insights into future perspectives about circRNAs in basic, translational and clinical research, eventually advancing the diagnosis, prevention and treatment of HCC.

## 1 Introduction

Liver cancer, the leading cause of cancer-related deaths in many countries, remains a global health challenge (Akinyemiju et al., 2017), and its prevalence continues to increase with an estimated incidence of >1 million cases by 2025 (Llovet J. et al., 2021). Hepatocellular carcinoma (HCC) represents the most frequent primary liver cancer. Most HCC patients are usually diagnosed at an advanced stage and accompanied by metastasis. In this regard, early detection is an effective option to attenuate HCC-related death and achieve long-term disease-free survival. Unfortunately, the only blood-based biomarker currently validated for HCC surveillance is α-fetoprotein. Furthermore, the principal cancer diagnosis methods, including imaging examination and histopathology (Yang and Heimbach, 2020), are difficult to detect concealed early symptoms of HCC. Therefore, early diagnosis of HCC remains a challenge that needs to be solved. On the other hand, researchers have been committed to finding effective systemic management for HCC for the past 70 years. Although the treatment of HCC is becoming more reasonable, the curative effect has not reached the ideal impact. For example, Sorafenib and Lenvatinib, two representative first-line drugs for advanced HCC, are just effective in a subset of patients. In addition, locoregional therapies play a substantial role in the management of 50%–60% HCC, but molecular therapies dominate the adjuvant trials after curative therapies (Llovet et al., 2021b). Thus, the molecular mechanisms underlying HCC pathogenesis, especially the identification of new druggable targets, are of significance for the prevention and treatment of the HCC pandemic.

**FIGURE 1:**
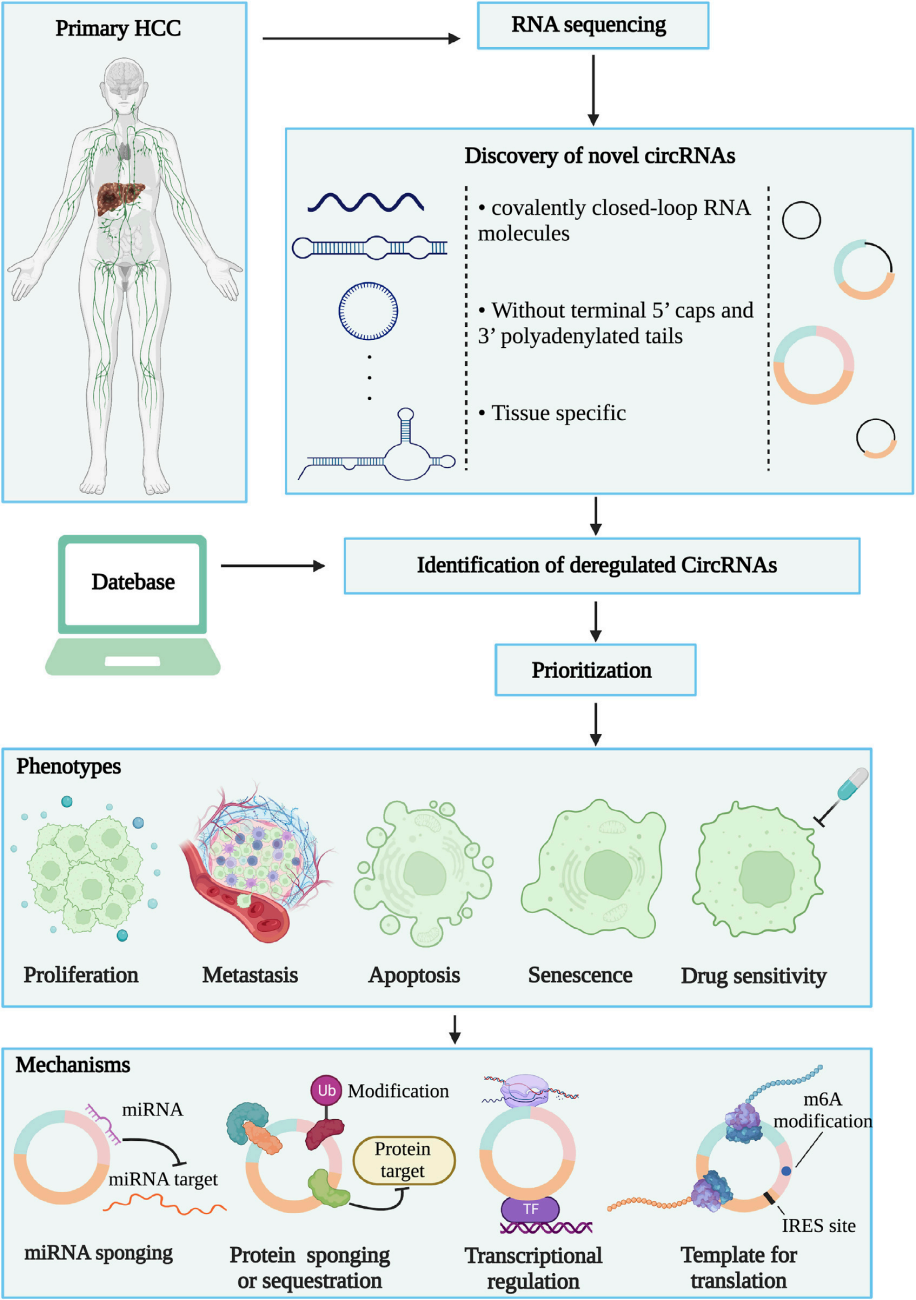
Identification and research strategies of circRNAs. Firstly, RNA sequencing is performed between hepatocellular carcinoma tissue and normal liver tissue. By comparing the two sets of sequencing data, novel or deregulated circRNAs could be found. Then, the technology of omics data integration and bioinformatics analysis prioritized circRNAs candidates. Finally, the screened circRNAs will be disclosed the function and mechanism characterization of liver cancer through biological experimental technology.

CircRNAs, as the name suggests, are a class of single-stranded closed circle molecules that lack 5’ and 3’ ends and poly (A) tails, which make them resistant to RNase R and more stable than linear RNAs (Liu and Chen, 2022). When first discovered in the early 1970 s, circRNAs were thought to be byproducts of splicing without meritorious biological functions (Hsu and Coca-Prados, 1979). However, with the emergence of high-throughput sequencing technologies and bioinformatics approaches, many differentially expressed circRNAs have been identified in viruses, insects, plants and mammals (Tholken et al., 2019; Zhang P. et al., 2020; Espindola et al., 2021; Jiang et al., 2021). Moreover, numerous studies have indicated the potential of circRNAs as a promising disease biomarker. A common experimental standard for identifying, isolating, analyzing circRNAs is essential for circRNA biology. The improvement of practice processes and standards also provides convenience for circRNA research (Nielsen et al., 2022) (Figure 1). Certainly, increasing evidence suggests that circRNAs are involved in the occurrence and development of various diseases (Gomes et al., 2020; Stoll et al., 2020). In particular, most studies have focused on the role of circRNAs in cancers and demonstrated their pivotal roles in the pathogenesis of cancer (Wang Y. et al., 2020; Wang Y. et al., 2022), including HCC (Yang X. et al., 2021; Chen and Shan, 2021). Indeed, many classical tumor-related factors and signaling pathways, such as c-Myc, MAPK and Hedgehog, are mediated by circRNAs in HCC (Hu et al., 2020; Gu et al., 2021b; Chen et al., 2022b). These results indicate that circRNAs are emerging as novel cancer biomarkers and therapeutic targets.

In recent years, tremendous efforts have been devoted to unraveling the involvement of circRNAs in HCC pathology. These exciting advances had brought new insights into tumorigenesis, and provided alternative tools for diagnosis and potential therapeutic approaches in clinic for HCC. In this review, we summarized the characterization, roles, and underlying mechanisms of circRNAs in HCC, and also discussed the current dilemma and future development direction of circRNAs.

## 2 Non-coding RNAs

In the nucleus of human cells, approximately 90% of the genomic sequence is transcribed into RNA, but less than 2% can be translated into functional proteins. The rest untranslated portion is traditionally regarded as “dark matter” that contains a vast portion of functional non-coding RNAs (ncRNAs), as demonstrated by the ENCODE project (Doolittle, 2013). NcRNAs can be grouped into several classes by size. Small ncRNAs (sncRNAs), including microRNAs (miRNAs), Piwi-interacting RNAs (piRNAs) and tRNA-derived small RNAs (tsRNAs), play a key role in carcinogenesis. Long ncRNAs (lncRNAs), comprising ncRNAs longer than 200 nucleotides (nt), include pseudogenes and circRNAs (Slack and Chinnaiyan, 2019). NcRNAs are involved in numerous physiological and pathological processes, and thus modulate disease progression (Fu et al., 2015; Tian et al., 2018; Ma et al., 2019; Zhang W. et al., 2021). Accordingly, the recent decades have witnessed remarkable advances of ncRNAs, particularly miRNAs and lncRNAs, in both physiology and pathology (Liu G. et al., 2020; Du W. et al., 2022; Zou et al., 2023a). Although most of the molecular mechanisms are incompletely clear, circRNAs have shown universal and critical roles in regulating cellular processes of various diseases including cancers, which emphasizes the importance of circRNAs (Li et al., 2020b).

## 3 CircRNAs

CircRNAs, a class of ncRNAs, are usually generated from fragments of linear pre-messenger RNAs or other linear RNA species (Liu and Chen, 2022). CircRNAs are a kind of tissue-specific and covalently closed-loop RNA molecules, which are different from other ncRNAs (Liu and Chen, 2022). CircRNAs, which are without terminal 5’ caps and 3’ polyadenylated tails, are more stable than other ncRNAs. This high stability may be due to its covalently circular structure that avoids exonuclease-mediated degradation (Hou et al., 2023). The mechanisms underlying circRNA biosynthesis remain unclear. Currently, there are two patterns of circRNAs formation, namely, the ‘direct back-splicing’ model and the ‘lariat intermediate’ model (Chen L., 2020). According to the biogenesis mechanisms, four types of circRNAs have been classified: exonic circRNAs (ecircRNAs), intronic circRNAs (ciRNAs), exon-intron circRNAs (EIciRNAs), and intergenic circRNAs (icircRNAs). EcircRNAs are composed of one or several exons of cyclization, whereas ciRNAs contain only introns. Exons and introns cross cyclization are the existence form of EIciRNAs. Approximately 84% of circRNAs are derived from protein-coding genes (van Zonneveld et al., 2021), however, the region of icircRNAs sequency between two protein-coding genes. EcircRNAs and intergenic circRNAs are located in the cytoplasm, while ciRNAs and EIciRNAs generally localize to the nucleus. By the way, ecircRNAs make up the majority of circRNAs and are more conserved than other types of circRNAs (Liu and Chen, 2022). Recently, Shan et al. discovered a new class of circRNAs, named mecciRNAs, which are encoded by the mitochondrial genome (Liu et al., 2020c). MecciRNAs can facilitate the mitochondrial entry of nuclear-encoded proteins by serving as molecular chaperones. Further studies found that circRNAs are involved in immune responses, cell proliferation and transformation, and neuronal functions (Yan and Chen, 2020; D'Anca et al., 2022). In addition, dysregulation of circRNAs is associated with a wide range of diseases and can have phenotypes in animal models.

### 3.1 Biogenesis and regulation of circRNAs

The formation of circRNAs is different from other RNAs. CircRNAs are derived from canonical 3’ and 5’ splice sites (Vo et al., 2019). High-throughput analyses of genomic characterization of circRNAs exons combined with mutational analyses in circRNA expression vectors showed that the canonical spliceosomal machinery carries out a particular type of alternative splicing, named back-splicing (Starke et al., 2015). CircRNAs are generated from linear pre-messenger RNAs and other linear RNA species through back-splicing. This particular type of splicing connects the RNA downstream 5’ splice-donor (SD) site and an upstream 3’ splice-acceptor (SA) site to form a covalently closed loop (Dragomir and Calin, 2018; Chen L. L., 2020). CircRNAs usually have long introns on the flanks of exons, and back-splicing mediates the base-pairing of introns by inverted repeat elements (such as Alu elements) and looping formation (Jeck et al., 2013; Zhang X. O. et al., 2014). The base-pairing between inverted repeat elements recruits a downstream SD site into proximity with an upstream SA site, then an upstream branch point (BP) attacks a downstream SD site. After 5’ end of a downstream SD site breaks away from pre-mRNA, the downstream SD site attacks an upstream SA site and binds closely (Kristensen et al., 2019; Long et al., 2021). This connection results in the formation of EIciRNAs or ecircRNAs (the internal intron is spliced out). In addition, the dimerization of RNA-binding proteins (RBP) such as protein quaking, FUS, HNRNPL and MBL, has been shown to regulate certain circRNAs biogenesis by binding to specific elements in flanking introns (Yang T. et al., 2021; Ho et al., 2021; Han J. et al., 2022; Pamudurti et al., 2022).

In addition to the biogenesis model mentioned above, a ‘lariat intermediate’ model has been purposed, in which canonical linear splicing happens first to produce a linear mRNA and long intron lariat (Liu and Chen, 2022). CiRNAs could derive from intron lariat and escape the debranching step of linear splicing (Zhang et al., 2013). More importantly, exon-skipping events usually occur during linear splicing and produce a long intron lariat. The long intron lariat containing the skipped exon can mediate the formation of circRNA by internal back-splicing (Kelly et al., 2015; Eger et al., 2018). Notably, not every exon-skipping event can trigger back-splicing to produce circRNAs, but skipping exons that undergo back-splicing are less present in mature mRNAs (Kelly et al., 2015). Although the above mechanism hypotheses have been widely studied, the precise mechanisms of circRNA biogenesis are still obscure.

Both models rely on back-splicing, which is accomplished by the canonical splicing machinery. In this regard, anything modulating the spliceosome might logically influence the formation of circRNAs. Using RNAi screening in *Drosophila melanogaster*, Liang et al. found that depletion of SF3b or SF3a, components of the U2 small nuclear ribonucleoprotein particle (snRNP) that are required for pre-spliceosome assembly, resulted in obviously increased in circRNAs levels, accompanied by decreased linear RNA levels (Liang et al., 2017). This phenomenon suggests that back-splicing becomes a preferred pathway of pre-mRNA processing when canonical pre-mRNA processing events are impaired (Liang et al., 2017). Furthermore, some spliceosomal factors are involved in circRNA formation through directly or indirectly binding to specific RNA motifs. For example, Quaking (QKI) strengthens circRNA formation by binding to specific sites in flanking introns (Conn et al., 2015). The YY1 complex could bind to the super-enhancer and promoter of QKI to increase the formation of certain circRNAs (Han J. et al., 2022). Kramer et al. found that the combination of *cis*-acting elements and *trans*-acting splicing factors, including hnRNP (heterogeneous nuclear ribonucleoprotein) and SR (serine–arginine) proteins, regulates the biogenesis of Laccase circRNA (Kramer et al., 2015). RBPs, except core spliceosomal factors, can modulate back-splicing in *trans*-acting by binding the intronic complementary sequence (ICS). NF90 and NF110, both of which are protein products of interleukin enhancer-binding factors 3 (ILF3) and involved in the host antiviral system, are able to promote circRNA production by acting on inverted-repeat Alu elements and stabilizing intronic RNA pairs in the nucleus (Li et al., 2017; Shang et al., 2022). In contrast to NF90 and NF110, double-stranded RNA-specific adenosine deaminase 1 (ADAR1) prevents the looping of flanking intron by performing adenosine to inosine editing. Accordingly, the destruction of inverted-repeat Alu elements is capable of impairing the complementarity and stability of intronic RNA pairs (Ivanov et al., 2015; Eisenberg and Levanon, 2018). Similar as ADAR1, DHX9 is an ATP-dependent nuclear RNA helicase and can suppress circRNA biogenesis by binding to inverted-repeat Alu elements and unwinding RNA pairs that flank circularized exons (Aktas et al., 2017). Interestingly, DHX9 is remarkably upregulated in HCC tissues and regulates the expression of circDLC1, which inhibits liver cancer progression (Liu H. et al., 2021). In addition, Zhang et al. found that back-splicing is correlated with RNA Polymerase II transcription elongation rate (TER). The faster TER of genes results in a higher probability and production of circRNAs (Zhang et al., 2016), which probably due to the fact that a higher TER could transcribe more downstream ICS, thereby increasing the possibility of ICS pairing across exons (Chen L. L., 2020).

### 3.2 Biological functions of circRNAs

**FIGURE 2:**
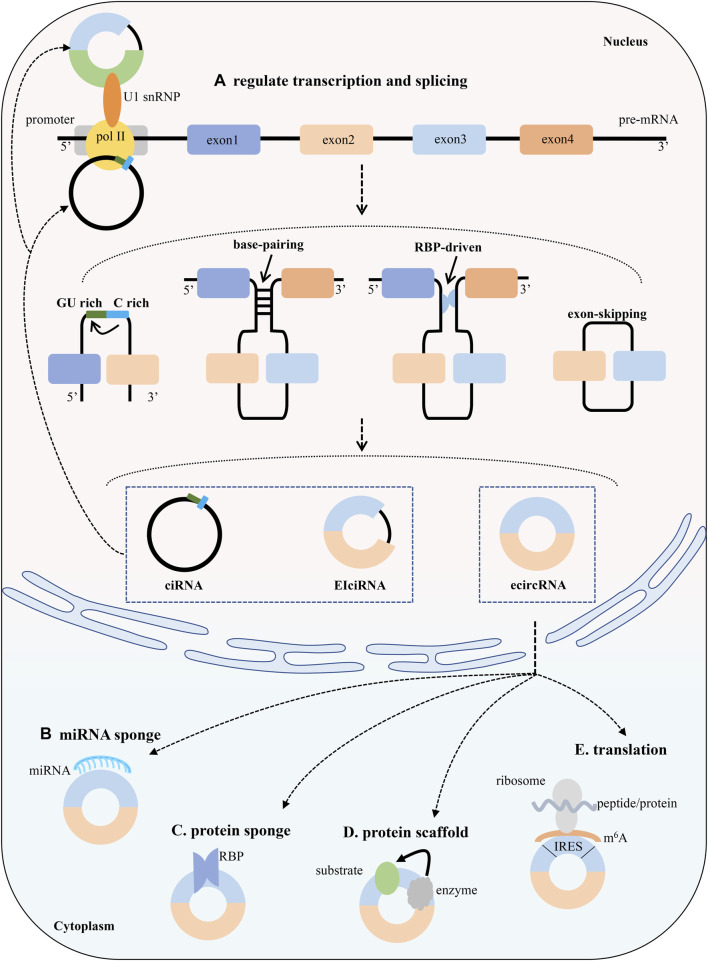
Biogenesis and functions of circRNAs. The canonical back-splicing generates three types of circRNAs: ciRNAs, EIciRNAs and ecircRNAs. CircRNAs enhance the transcription and splicing of their parental genes by interacting with RNA pol II or U1 small nuclear snRNP. Functions **(A)** Competition with splicing and regulation of parental gene transcription **(B)** miRNA sponges. CircRNAs affects the expression of miRNA and downstream target genes by adsorbing miRNAs **(C)** Protein sponges. CircRNAs regulates target proteins by binding to them **(D)** Prontein scaffolds. CircRNAs facilitates the formation of a complex **(E)** Translation. CircRNAs can be translated into peptides or proteins.

Truth will not be changed by misunderstanding of people. CircRNAs initially existed in the world as by-products of splicing, nevertheless, accumulating data demonstrate that circRNAs have diverse biological functions, such as miRNAs sponge and proteins scaffold. Figure 2 shows the function of circRNAs, and the further details are described as follows.

#### 3.2.1 CircRNAs can act as miRNA sponges

The great majority of circRNAs are predominantly localized in the cytoplasm and contain miRNA-response elements. Consequently, circRNAs can function as competing for endogenous RNAs (ceRNAs) or miRNA sponges to sequester miRNA, and thus prevent the interactions between miRNA and mRNA (Cui et al., 2021). CDR1as (cerebellar degeneration-related protein 1 antisense transcript; also known as ciRS-7) is the most representative circRNA as yet, containing more than 70 conversed binding sites for miR-7. Thus, CDR1as can function as a negative regulator of miR-7, leading to an increase in the expression of miR-7 target genes (Guo et al., 2020). Intriguingly, CDR1as and miR-7 are enriched in brain tissues (Memczak et al., 2013), indicating that CDR1as may be involved in the development and function of the nervous system. Indeed, CDR1as-knockouted mice exhibited neuropsychiatric disorders associated with a strong deficit in prepulse inhibition of the startle response (Piwecka et al., 2017). In addition to its neuron-modulating function, CDR1as has been recently shown to regulate tumorigenesis, including HCC, through sponging miR-7. For example, Yang et al. found that CDR1as promotes HCC cell proliferation at least in part through the regulation of the expression of epidermal growth factor receptor (EGFR), a target gene of miR-7 (Yang X. et al., 2017). Other miR-7 target genes, such as phosphoinositide 3-kinase catalytic subunit delta (PIK3CD) and cyclin E1 (CCNE1), may also attribute to the oncogenic role of CDR1as in HCC progression (Fang et al., 2012; Zhang X. et al., 2014). Consistently, CDR1as is also upregulated and facilitates cell proliferation by activating miR-7 target genes in esophageal cancer, osteosarcoma, and nasopharyngeal carcinoma (Li R. et al., 2018; Xu et al., 2018; Zhong et al., 2019). Beyond CDR1as, CircZNF91 is regarded as another representative miRNA sponge. It contains 24 target sites for miR-23b-3p, a crucial regulator of many physiological processes, providing an attractive target for stem cell biology (Zeng Z. et al., 2021). The mouse circSry, derived from sex-determining region Y, contains 16 target sites for miR-138-5p and is involved in the development of testis (Song et al., 2023). These studies suggest that there is a competitive relationship between circRNAs and mRNA in binding miRNA. Of note, unlike the above-mentioned circRNAs that can act as a miRNA sponge, the vast majority of circRNAs do not contain more miRNA binding sites than expected by chance (Guo et al., 2014). Thus, when identifying the sponge role of circRNAs, it is necessary to consider the stoichiometric relationship between the miRNA binding sites of the circRNA and the mRNA target sites of the miRNA (Jarlstad Olesen and Lasse, 2021).

#### 3.2.2 CircRNAs interact with RBPs

CircRNAs can also interact with RBPs to modulate the function and location of associated proteins, functioning as either protein scaffolds or protein sponges. Du et al. conducted a series of studies on circ-Foxo 3, a circular RNA generated from a member of the forkhead transcription factors family (Du et al., 2016). These studies demonstrate a role of Circ-Foxo 3 in cell cycle progression. Specifically, ectopic expression of circ-Foxo3 in the cytoplasm can repress cell cycle progression by binding to 2 cell cycle proteins, cyclin-dependent kinase 2 (CDK2) and cyclin-dependent kinase inhibitor 1 (p21), resulting in the formation of a ternary complex that prevents disease progression, such as HCC. Cellular senescence is defined as the arrest of cell cycle progression. Interestingly, circ-Foxo 3 can interact with several proteins, including the anti-senescent protein ID-1, the transcription factor E2F1 and the anti-stress proteins FAK and HIF1α, which subsequently prevents FAK to locate to mitochondria or HIF1α to translocate to the nucleus, exacerbating cardiac cell senescence under stressed conditions (Du et al., 2017b). In addition, circ-Foxo 3 promotes the mouse double-minute 2 (MDM2)-mediated ubiquitylation of mutant p53, and facilitates the proteasome-meditated mutant p53 degradation in breast carcinoma (Du et al., 2017a).

It has shown that a number of circRNAs could bind to HuR, a crucial RBP regulator for protein translation, and thus play a role in tumorigenesis. For instance, circPPFIA1s are generated from PPFIA1 with different length, including circPPFIA1-L (long) and -S (short). CircPPFIA1s binds to HuR and destroys the mRNA stability effect of HuR on oncogenic RAB36, thus decreasing RAB36 expression and inhibiting the liver metastasis of colorectal cancer (Ji et al., 2022). CircBACH1 is able to directly interact with HuR and facilitate HuR translocation to the cytoplasm, resulting in decreased p27 translation and increased HCC cell proliferation (Liu B. et al., 2020). Interestingly, recent studies suggest that circRNAs might serve as an entry point for tumor immunotherapy. It has shown that endogenous circRNAs tend to form a 16–26 bp imperfect RNA duplexes and bind to double-stranded RNA (dsRNA)-activated protein kinase (PKR), and thus prevent aberrant activation of innate immunity (Liu C. X. et al., 2019). In response to poly (I:C) stimulation or viral infection, circRNAs are globally degraded by RNase L, and PKR molecules are released to activate the immune response. It is of interest for future investigation to delineate the interplay of circRNAs and their associated proteins in immunity and tumorigenesis.

#### 3.2.3 CircRNAs regulate transcription and splicing

A few circRNAs are localized in the nucleus and play a role in transcription regulation. CircRNAs in the nucleus, mainly CiRNA and EIciRNA, are involved in gene expression at the transcription level (Zhang et al., 2013; Li Z. et al., 2015). CircRIG-I binds to DDX3X and activates MAVS/TRAF5/TBK1 signaling cascade, which are crucial for activation of host innate immunity. TBK activates the transcriptional factor IRF3 through phosphorylation to form homodimers, eventually initiating the transcription of type I IFNs and various antiviral genes *via* binding to the target genome region (Song et al., 2022). On the contrary, several circRNAs can inhibit the transcription of their target genes. Circ-HuR directly interacts with CCHC-type zinc finger nucleic acid binding protein (CNBP) and acts as an inhibitor to restrain the binding of CNBP to HuR promoter, resulting in HuR repression (Yang F. et al., 2019). Notably, recent studies have indicated that several cytoplasmic circRNAs are also involved in the modulation of transcription. For example, circSLC25A16 in the cytoplasm can promote the transcription of lactate dehydrogenase A (LDHA) by interacting with miR-488-3p and hypoxia-inducible factor 1-alpha (HIF-1α) (Shangguan et al., 2020). CircPPKAA1 recruits mSREBP-1 and Ku80/Ku70 to form a ternary complex in HCC, thus facilitating the stability of mSREBP-1, a transcription factor to drive expression of essential fatty acid synthesis enzymes. Of note, the circPPKAA/Ku80/Ku70/mSREBP-1 ternary complex locates to the promoters of the fatty acid biosynthesis genes, and upregulates their transcription, including ACC1, ACLY, FASN and SCD1 (Li Q. et al., 2022). Although a growing number of circRNAs have been identified to be involved in the transcription of their host genes, the relevant mechanisms of action remain unclear.

Notably, circRNAs also influence the biogenesis of itself through affecting the canonical splicing and host gene transcription. Working in *Drosophila* indicated a genome-wide competition between canonical splicing and circRNAs generation. For example, MBL promotes the circularization of its own exon 2 to the detriment of mRNA production (Ashwal-Fluss et al., 2014). Another study, by exhausting or inhibiting the spliceosome components, found that the steady-state levels of circular RNAs were increased while the expression of their associated linear mRNAs concomitantly decreased (Liang et al., 2017). Back-splicing and canonical splicing use the same 3’ and 5’ splice sites may be part of the reasons for the negative correlation between mRNA production rates and circRNA biogenesis rates.

#### 3.2.4 CircRNAs can translate proteins or peptides

CircRNAs were initially considered as a kind of endogenous ncRNA, since they lack the typical 5’ cap and 3’ poly(A) tail that are the essential elements for cap-dependent translation initiation. Nevertheless, a vast part of circRNAs are mainly located in the cytoplasm, the location of translation, and derived from the middle exons of pre-mRNA. More importantly, cap-independent translation pattern has been discovered (Wang J. et al., 2022). This indicates translation may be the potential function of circRNAs. As expected, a growing body of research has shown that translation can also proceed on circRNAs through cap-independent way, namely, internal ribosome entry sites (IRESs) or N6-methyladenosine (m^6^A)-dependent translation (Wen et al., 2022). Circular E-cadherin (circ-E-Cad) RNA, which is obviously enriched in glioblastoma, contains a potential IRES. IRES may have the potential possibility to mediate cross-junction and multiple-round ORF (open reading frame) to encode a 254-amino acid protein, named circRNA-encoded E-cadherin (C-E-Cad) (Gao et al., 2021). In addition, circMbl in *Drosophila* head extracts, circZNF609 in myogenesis, circPINTexon2, circSHPRH and circFBXW7 in glioma tumorigenesis, all of them are IRES-mediated protein-coding circRNAs (Legnini et al., 2017; Zhang et al., 2018a; Zhang et al., 2018b; Yang et al., 2018; Pamudurti et al., 2022). Of course, IRES-driven protein-coding circRNAs also exist in liver cancer. For example, circβ-catenin encodes a new 370-amino acid isform of β-catenin depending on its own existence IRES sequence (Liang et al., 2019). The β-catenin isoform binds to GSK3β protein kinase, thereby antagonizing the GSK3β-mediated ubiquitination degradation of β-catenin and activating the Wnt pathway. Notably, several IRES-activated protein-coding circRNA, such as circZNF609, at the same time with high m^6^A methylation levels, which indicates that there may be an unclear association between the two cap-independent translation ways (Zhao et al., 2014; Legnini et al., 2017). However, whether there are other cap-independent ways to initiate circRNA translation remains unclear. Given that proteins are the exerciser and undertakers of cell activities, the next step is still essential to determine the function of the encoded proteins. Indeed, several studies have found that cells undergoing heat stock and starvation conditions can alter circRNA translation, indicating that circRNA-encoded proteins may play roles in stress response (Pamudurti et al., 2017).

## 4 CircRNAs in HCC

The liver is the largest solid organ in mammals, and is considered to be the busiest organ in the body due to its many functions. Of note, the function of the liver in regulating energy homeostasis is particularly important, which executes its anabolic or catabolic programs to balance nutrient metabolism. The stability of liver metabolic function is essential for body homeostasis, and dysfunction of metabolism will lead to the occurrence of liver disease, including HCC (Ma et al., 2022). As a rising star for the past few years, circRNAs have been proven to be involved in the physiological function of liver. High-throughput sequencing has identified many circRNAs, such as circ_0001452, circ_0001453 and circ_0001454, which participate in hepatic lipid metabolism (Xie et al., 2022). Glucose metabolism is also an important manifestation of liver function. For example, circ_0000660 targets lgfbp1, an energy metabolism-related gene, and regulates glucose and lipid metabolism (Zeng Y. et al., 2021). Thus, circRNAs are closely related to the functional homeostasis of the liver, and the dysregulation of circRNAs may lead to liver disease.

**FIGURE 3:**
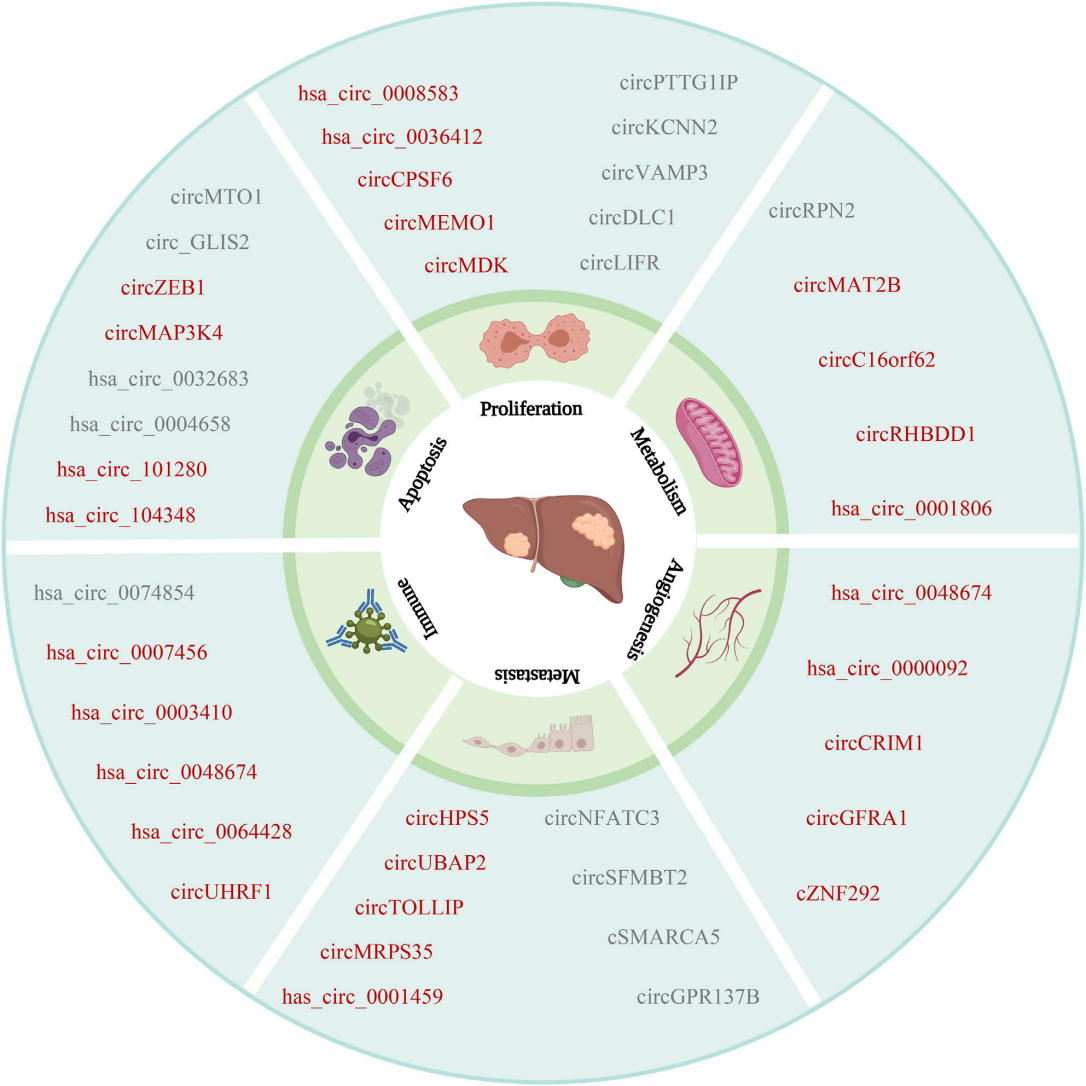
The relationship between circRNAs and HCC. CircRNAs are associated with the hallmarks of HCC. Including proliferation, metabolism, angiogenesis, metastasis, immune and apoptosis (The red font indicates oncogene, and the gray font indicates suppressor).

In fact, increasing evidence indicates that evolutionarily conserved ncRNAs, including circRNAs, play an important role in various pathological and physiological processes. To date, some circRNAs have been implicated in several hallmarks of cancer, such as cell death and survival, invasion, metastasis, and angiogenesis. Various validation tests, such as Northern blot, dot-blotting, RNA-seq and circRNA-specific microarrays, demonstrate that many key circRNAs are significantly dysregulated in HCC cells (Figure 3), tissues, blood and exosomes. Despite these recent progresses, the role of circRNAs in the occurrence and development of HCC is still in their infancy. There is no doubt that better understanding of circRNAs biology is valuable to further deepen the pathogenesis of HCC and develop novel targeted treatments.

### 4.1 CircRNA expression profile in HCC

With the development of high-throughput sequencing technology, genome-wide circRNA expression profiles in liver cancer have long been revealed. Sunagawa et al. found that seven candidate circRNAs were downregulated in HCC tumors compared with normal liver tissues (Sunagawa et al., 2021). By sequencing mononuclear cells from the peripheral blood of healthy people and HCC, a total of 58 circRNAs were found to be significantly changed (≥2 or ≤ 0.5-fold) (Lei et al., 2019). In addition, multiple circRNA-related databases also showed that circRNA expressions are substantially different between human HCC and cancer-free liver tissues, suggesting that the dysregulation of circRNAs may be involved in HCC (Rebolledo et al., 2023) (Table 1). Both circHIPK3 and circTEME45A are significantly upregulated in HCC cells and enhance cell proliferation, migration, and invasion (Zheng et al., 2016; Zhang T. et al., 2020). Of note, the same circRNA may have different roles in distinct cancers. For example, ciRS-7 promotes the proliferation and invasion of HCC cells by targeting miR-7, yet acts as a tumor suppressor in ovarian and bladder cancer (Yang X. et al., 2017; Chen et al., 2019b). The dysregulation of circRNAs suggests that they have emerged as a potential target for diagnosing HCC and therapeutic intervention.

**TABLE 1:** Relevant examples of circular RNAs and their related implications in HCC.

circRNAs	Role in HCC	Cancer pheotype	Target/Pathway	PMID	References
hsa_circ_0020007 (Circ-ADD3)	Tumor suppressive	Migration, invasion, metastasis	EZH2, CDK1	31,497,351	Sun et al. (2019)
Has-circ-0000567 (circSETD3)	Tumor suppressive	Proliferation	miR-421/MAPK14	30,795,787	Xu et al. (2019)
hsa_circRNA8662-12 (circTRIM33-12)	Tumor suppressive	Proliferation, migration, invasion	miR-191/TET1	31,153,371	Zhang et al. (2019c)
circADAMTS13	Tumor suppressive	Proliferation	miR-484/ADAMTS13	30,537,115	Qiu et al. (2019)
hsa_circ_0018665 (circADAMTS14)	Tumor suppressive	Proliferation, invasion	miR-572/RCAN1	30,317,540	Song et al. (2019)
hsa_circ_0000847 (circSMAD2; hsa_circSMAD2_005)	Tumor suppressive	Migration, invasion, EMT	miR-629	29,844,683	Zhang et al. (2018c)
circLP4	Tumor suppressive	Proliferation	miR-761/RUNX3/p53/p21	30,520,539	Chen et al. (2019c)
hsa_circ_001013 (circHIAT1)	Tumor suppressive	Growth	miR-3171/PTEN	31,108,351	Wang et al. (2019e)
hsa_circ_0001727 (hsa_circZKSCAN1_005; circZKSCAN1)	Tumor suppressive	Growth, migration, invasion	FMRP-CCAR1-Wnt signaling	31,281,495	Zhu et al. (2019b)
circ-0079929	Tumor suppressive	Growth	PI3K/AKT/mTOR signaling	30,655,696	Zheng et al. (2019)
circNFATC3	Tumor suppressive	Proliferation, migration, invasion	miR-548I/NFATC3/JNK/c-Jun/AKT/mTOR	32,667,692	Jia et al. (2021)
circC3P1	Tumor suppressive	Proliferation, migration, invasion	miR-4641/PCK1	29,608,893	Zhong et al. (2018)
hsa_circ_0091570	Tumor suppressive	Proliferation, migration	miR-1307/ISM1	31,207,319	Wang et al. (2019d)
hsa-_circ_0001649 (circSHPRH_019)	Tumor suppressive	Proliferation, migration, invasion, apoptosis (+)	miR-127-5p, miR-612, miR-4688/SHPRH	31,137,016	Su et al. (2019b)
circMTO1 (hsa_circ_0007874)	Tumor suppressive	Proliferation, invasion, apoptosis (+)	miR-9/p21	28,520,103	Han et al. (2017)
		Apoptosis (+)	miR-541-5p/ZIC1/Wnt/β-catenin pathway	34,930,906	Li et al. (2021c)
hsa_circ_0001445 (cSMARCA5; hsa_circSMARCA5_013)	Tumor suppressive	Proliferation, migration, growth, metastasis, apoptosis (+)	miR-17-3p, miR-181b-5p/TIMP3	29,378,234	Yu et al. (2018)
hsa_circ_0004018	Tumor suppressive	Proliferation, migration	miR-626/DKK3/Wnt/β-catenin signaling	33,061,423	Zhu et al. (2020c)
circ-0051443	Tumor suppressive	Proliferation, migration, growth	miR-331-3p/BAK1	32,014,458	Chen et al. (2020a)
circCDK13	Tumor suppressive	Migration, migratory, invasion	JAK/STAT, PI3K/ATK signaling	29,658,568	Lin et al. (2018)
has_circ_0000204	Tumor suppressive	Proliferation	miR-191/KLF6	31,334,580	Tian et al. (2019)
circLIFR	Tumor suppressive	Proliferation, metastasis	miR-624-5p/GSK-3β/β-catenin pathwat	35,581,180	Yang et al. (2022a)
circITCH (hsa_circ_0001141)	Tumor suppressive	Apoptosis (+)	miR-184	35,400,275	Guo et al. (2022)
hsa_circ_0098181	Tumor suppressive	Apoptosis (+)	miR-18a-3p/PPARα pathway	35,264,957	Luo et al. (2022)
circEPS15	Tumor suppressive	Proliferation, metastasis	TJP1/CDH2/VIM	35,211,158	Jiang et al. (2022)
circVAMP3	Tumor suppressive	Proliferation, metastasis	c-Myc pathway	35,072,355	Chen et al. (2022b)
circKCNN2	Tumor suppressive	Proliferation, colony formation, migration	miR-520c-3p/MBD2	35,051,313	Liu et al. (2022a)
circRPN2	Tumor suppressive	Metastasis, metabolism	ENO1/AKT/mTOR; miR-183-5p/FOXO1	35,045,986	Li et al. (2022a)
hsa_circ_0032683	Tumor suppressive	Proliferation, apoptosis (+)	miR-338-5p/RTN4	35,030,979	Shen et al. (2022b)
hsa_circ_0004658	Tumor suppressive	Proliferation, apoptosis (+)	miR-499b-5p/JAM3 pathway	35,013,102	Zhang et al., (2022)
circCCNB1	Tumor suppressive	Proliferation, metastasis	miR-106b-5p/GPM6A, DYNC1I1/AKE/ERK pathway	35,002,514	Liu et al. (2022g)
circGPR137B	Tumor suppressive	Proliferation, metastasis	miR-4739/FTO	35,858,900	Liu et al. (2022d)
circPTTG1IP	Tumor suppressive	Proliferation, metastasis	miR-16-5p/RNF125/JAK1 signaling	35,710,093	Peng et al. (2022)
circ_GLIS2	Tumor suppressive	Apoptosis (+)	EIF4A3	34,743,403	Xing et al. (2022)
circUBE2J2	Tumor suppressive	Proliferation, migration	miR-370-5p/KLF7	34,686,662	Zhang et al. (2021a)
hsa_circ_0074854	Tumor suppressive	Migration, invasion, immune	HuR	33,880,025	Wang et al. (2021d)
circARPP21	Tumor suppressive	Proliferation, invasion, migration	miR-543/LIFR	33,584,097	Gu et al. (2021b)
circZNF609	Oncogenic	Proliferation, metastasis, stemness	miR-15a-5p/15b-5p, GLI2/Hedgehog pathway	32,398,664	He et al. (2020b)
circ-LRIG3 (hsa_circ_0027345)	Oncogenic	Proliferation, migration, invasion, apoptosis (−)	EZH2/STAT3	33,222,697	Sun et al. (2020b)
circSOD2	Oncogenic	Growth, migration	miR-502-5p/DNMT3a/JAK2/STAT3/circSOD2	33,234,142	Zhao et al. (2020a)
circASAP1 (hsa_circ_0085616)	Oncogenic	Proliferation, invasion, metastasis	miR-326/miR-532-5p-MAPK1/CSF-1 signaling	31,838,741	Hu et al. (2020)
CDR1as (hsa_circ_0001946)	Oncogenic	Proliferation, migration	miR-1270/AFP	31,581,132	Su et al. (2019a)
hsa_circ_100,338	Oncogenic	Migration, invasion	miR-141-3p/RHEB/mTOR signaling	31,157,168	Huang et al. (2019)
circ-CDYL	Oncogenic	Proliferation, growth	miR-892a,miR-328-3p/PI3K-AKT serine//β-catenin,NOTCH2 pathways	31,148,183	Wei et al. (2020b)
circ-DB	Oncogenic	Proliferation, growth	miR-34a/USP7/Cyclin A2 signaling	30,546,088	Zhang et al. (2019a)
hsa_circ_0005075	Oncogenic	Proliferation, migration, invasion	miR-335/MAPK1	31,054,187	Yang et al. (2019d)
circ_BIRC6	Oncogenic	Proliferation, migration, invasion, apoptosis (−)	miR-3918/Bcl2	30,931,701	Yang et al. (2019b)
circSLC3A2 (hsa_circ_0022587)	Oncogenic	Proliferation, invasion	miR-490-3p/PPM1F	30,470,261	Wang et al. (2018)
circPTGR1 (hsa_circ_0008043, hsa_circ_0003731, hsa_circ_0088030)	Oncogenic	Migration, invasion	miR449a-MET	30,630,697	Wang et al. (2019a)
circDYNC1H1	Oncogenic	Growth, proliferation, migration	miR-140-5p/SULT2B1	30,864,145	Wang et al. (2019f)
circMAT2B (hsa_circ_0074854)	Oncogenic	Proliferation, migration, invasion, growth, metastasis	miR-338-3p/PKM2	31,004,447	Li et al. (2019b)
hsa_circ_0000517	Oncogenic	Proliferation, migration, invasion, growth	miR-326/SMAD6	32,774,154	He et al. (2020a)
		Viability, colony formation, growth, apoptosis (−)	miR-1296-5p/TXND5	32,523,376	Zang et al. (2020)
hsa_circ_0007456	Oncogenic	Migration, invasion, immune	miR-6852-3p/ICAM-1	33,462,208	Shi et al. (2021)
circRHOT1 (hsa_circRNA_102,034)	Oncogenic	Growth, metastasis	NR2F6	31,324,186	Wang et al. (2019b)
circ-ZEB1.33	Oncogenic	Proliferation	miR-200a-3p/CDK6	30,123,094	Gong et al. (2018)
hsa_circRNA_104,718	Oncogenic	Proliferation, migration, invasion, metastasis, apoptosis (−)	miR-218-5p/TXNDC5	31,278,132	Yu et al. (2019)
circ-MYLK (hsa_circ_0002768)	Oncogenic	Proliferation, invasion, migration	miR-362-3p/Rab23	31,413,665	Li et al. (2019c)
		Proliferation, migration, invasion, apoptosis (−)	miR29a/KMT5C	32,904,604	Gao et al. (2020)
circβ-catenin	Oncogenic	Growth	Wnt pathway	31,027,518	Liang et al. (2019)
hsa_circ_SLAIN1 (hsa_circ_0100,929, hsa_circ_101,280)	Oncogenic	Proliferation, growth, apoptosis (−)	miR-375/JAK2	30,302,825	Cao et al. (2019)
circ-FOXP1	Oncogenic	Proliferation, invasion, apoptosis (−)	miR-875-3p, miR-421/SOX9	31,698,267	Wang et al. (2020)
hsa_circ_0046600	Oncogenic	Invasion, migration	miR-640/HIF-1α	31,807,009	Zhai et al. (2019)
hsa_circ_10720	Oncogenic	Invasion, metastasis	Twist/Cel/vimentin	29,844,124	Meng et al. (2018)
circFBLIM1	Oncogenic	Proliferation, invasion, apoptosis (−)	miR-346/FBLIM1	30,053,867	Bai et al. (2018)
SCD-circRNA 2	Oncogenic	Proliferation	ERK	31,235,426	Dong et al. (2019)
hsa_circ_0021093	Oncogenic	Growth, migration, invasion, apoptosis (−)	miR-766-3p/MTA3	31,330,234	Liu et al. (2019c)
circVAPA	Oncogenic	Proliferation	miR-377-3p/PSAP	31,368,365	Liu et al. (2019a)
circPTPRM	Oncogenic	Proliferation, migration, invasion	——	31,494,761	Luo et al. (2019b)
circ_IGF1R	Oncogenic	Proliferation, apoptosis (−)	PI3K/AKT	31,377,314	Fu et al. (2019)
circPVT1	Oncogenic	Proliferation, migration	miR-203/HOXD3	31,551,242	Zhu et al. (2019a)
circERBIN	Oncogenic	Proliferation	miR-1263/CDK6	35,530,358	Yang et al. (2022b)
circMDK (hsa_circ_0095868)	Oncogenic	Proliferation, migration, invasion	miR-346, miR-874-3p/ATG16L1/PI3K/AKT/mTOR pathway	35,524,319	Du et al. (2022a)
circTOLLIP	Oncogenic	EMT	miR-516a-5p/PBX3	35,509,064	Liu et al. (2022f)
circ_0000854	Oncogenic	Proliferation	miR-1294/IRGQ	35,417,749	Lin et al. (2022)
circ_0036412	Oncogenic	Proliferation	Hedgehog pathway	35,382,824	Wang et al. (2022b)
circRHBDD1	Oncogenic	Metabolism	YTHDF1/PIK3R1	35,317,519	Cai et al. (2022)
circ_ZEB1	Oncogenic	Proliferation, apoptosis (−)	miR-199a-3p/PIK3CA signaling	35,277,182	Liu et al. (2022e)
hsa_circ_0001394	Oncogenic	Proliferation, migration, invasion	miR-527/UBE2A	35,361,760	Yan et al. (2022)
hsa_circ_0048674	Oncogenic	Angiogenesis, immune, apoptosis (−)	miR-223-3p/PDL1	35,187,630	Li et al. (2022d)
circ_0001459	Oncogenic	Proliferation, migration, invasion	miR-6165/IGF1R	35,199,365	Shen et al. (2022a)
circ_0003945	Oncogenic	Proliferation, migration, invasion	miR-34c-5p/LGR4/β-catenin pathway	35,170,199	Lyu et al. (2022)
circCPSF6	Oncogenic	Proliferation, metastasis	PCBP2/YAP1	34,916,222	Chen et al. (2022d)
circFOXM1	Oncogenic	Proliferation, metastasis	miR-1179/SPAG5	34,903,799	Wang et al. (2021a)
circACTG1	Oncogenic	Proliferation, invasion, migration	miR-940/RIF1/AKT/mTOR	35,769,514	Wu et al. (2022)
circCRIM1	Oncogenic	Proliferation, angiogenesis	miR-378a-3p/SKP2	34,869,393	Ji et al. (2021)
hsa_circ_0003410	Oncogenic	Immune	miR-139-3p/CCL5	34,890,089	Cao et al. (2022)
hsa_circ_0058493	Oncogenic	Proliferation, metastasis	METTL3-hsa_circ_0058493-YTHDC1	34,888,309	Wu et al. (2021)
circIPO11	Oncogenic	Self-renewal	TOP1/GLI1/Hedgehog pathway	34,649,567	Gu et al. (2021a)
circMRPS35	Oncogenic	Proliferation, migration, invasion, clone formation, cell cycle	miR-148a-3p/STX3/PTEN	34,450,251	Li et al. (2022b)
circUBAP2	Oncogenic	proliferation, colony formation, migration, invasion	miR-1294/c-Myc pathway	34,314,478	Yu et al. (2021)
		proliferation, colony formation, migration, invasion	miR-194-3p/MMP9	34,239,873	Liu et al. (2021a)
circC16orf62	Oncogenic	Proliferation, invasion, metabolism	miR-138-5p/PTK2/AKT pathway	34,108,451	Zhang et al. (2021c)
hsa_circ_0004277	Oncogenic	Proliferation, migration, EMT	ZO-1	33,511,111	Zhu et al. (2020a)
circARNT2	Oncogene	Proliferation, apoptosis (−)	miR-155-5p/PDK1	33,425,483	Li et al. (2021c)
hsa_circ_104,348	Oncogene	Proliferation, migration, invasion, apoptosis (−)	miR-187-3p/RTKN2/Wnt/β-catenin	33,311,442	Huang et al. (2020b)
circMAP3K4	Oncogene	Apoptosis (−)	circMAP3K4-455aa/AIF	35,366,894	Duan et al. (2022)
circMET	Oncogene	Immune	miR-30-5p/Snail/DPP4/CXCL10	32,430,013	Huang et al. (2020c)
hsa_circ_0001806	Oncogene	proliferation, migration, metabolism	miR-125b/HK2	34,664,737	Chen et al. (2021a)

### 4.2 CircRNAs regulate HCC cell proliferation

To some extent, the ability to maintain chronic proliferation is the most fundamental characteristic of cancer cells. Cancer cells manipulate this ability by activating multiple oncogenic signaling pathways excessively. In mammals, many circRNAs promote HCC cell proliferation through activating oncogenic pathways, such as Wnt (Liang et al., 2019; Wei et al., 2020a), signal transducer and activator of transcription 3 (STAT3) (Chen L. et al., 2020; Sun et al., 2020b; Zhao Z. et al., 2020) and NOTCH (Wang L. et al., 2019; Wei Y. et al., 2020) signaling pathways. Deregulation of Wnt signaling has been linked to a number of human diseases, especially cancers. Circβ-catenin promotes HCC cell proliferation by encoding a 370-amino acid β-catenin isoform that can activate the Wnt pathway (Liang et al., 2019). Circ-DENND4C, an oncogene identified in breast cancer, was also overexpressed in HCC cells. It mediated malignant phenotypes in HCC cells through sequestering miR-195-5p, and thus augmented transcription factor 4 (TCF4) expression, leading to the activation of the Wnt/β-catenin signaling pathway (Liu et al., 2020d). Furthermore, several β-catenin downstream targets are also upregulated in liver cancer, such as C-MYC (Liang et al., 2019). Indeed, C-MYC often appears in the process of tumor occurrence and development (Dang, 2012). Hsa_circ_0091581 is derived from the GPC3 gene locus, and can regulate HCC progression by binding to molecules such as Wnt signaling proteins (Zhou F. et al., 2018). In addition, hsa_circ_0091581 promoted HCC cell proliferation *via* the hsa_circ_0091581/miR-526b/c-Myc axis (Wei et al., 2020a). The Janus kinase (JAK)/STAT signaling pathway is also involved in cancer cell migration, growth and differentiation (Leonard and O'Shea, 1998). CircSOD2, a circular RNA derived from SOD2 gene, promoted HCC cell growth by sponging miR-502-5p, thereby activating the DNMT3a/JAK2/STAT3 signaling axis (Zhao Z. et al., 2020). Circ-LRIG3, a novel covalently closed circRNA originated from the back-splicing of LRIG3 exon 2–11, can interact with EZH2 and STAT3 to form a ternary complex. The complex facilitates EZH2-induced STAT3 methylation and subsequent phosphorylation, and then activates STAT3 signaling and promotes HCC proliferation (Sun et al., 2020b). Of note, several circRNAs could maintain a specific cell state by multiple patterns. For example, circ-MALAT1, derived from lncRNA MALAT1, functions as a brake to form a complex with both ribosomes and mRNAs of paired box 5 (PAX5), a well-known tumor suppressor (Chen L. et al., 2020). The resultant tertiary complex retards PAX5 translation and promotes the self-renewal of HCC stem cells. In addition, circ-MALAT1 acts as a miR-6887-3p sponge to activate the JAK2/STAT3 signaling pathway, thus promoting HCC stem cell self-renewal. Circ-CDYL activates the PI3K-AKT-mTORC1/β-catenin and NOTCH2 pathways by sponging miR-892a and miR-328-3p respectively, which promote the translation of stem-cell–associated genes C-MYC and SURVIVIN and facilitate HCC tumorigenesis (Wei Y. et al., 2020). CircRHOT1 can accelerate the progression of HCC by recruiting TIP60 to the NR2F6 promoter, initiating NR2F6 transcription and eventually activating the NOTCH signaling pathway (Wang L. et al., 2019). NR2F6, a negative regulator of T cell development, is involved in human disease such as leukaemia and cancer (Klepsch et al., 2018; Malouf et al., 2021), albeit its involvement in HCC remains elusive. There is no doubt that many other signaling pathways might be involved in circRNA-mediated HCC cell proliferation. It is of great interest to expand the repertoire of circRNA-modulating signaling pathways in HCC tumorigenesis in the future.

In contrast, some circRNAs may inhibit HCC progression through inhibiting oncogenic pathways, such as Wnt (Zhang et al., 2019c) and STAT3 (Yu et al., 2018). TET1 is a member of the ten elven translocation (TET) family of methylcytosine dioxygenases, which participate in DNA demethylation by converting 5-methylcytosine (5mC) to 5-hydroxymethylcytosine (5hmC) (Tahiliani et al., 2009; Fu et al., 2013). Downregulated 5hmC/TET1 or upregulated 5 mC has been shown to be related with HCC progression (Chuang et al., 2015). Intriguingly, circMEMO1 is significantly reduced in HCC tissue and its expression is reversely associated with poor patient prognosis. Mechanistically, circMEMO1 could act as a sponge for miR-106b-5p and then modulate the promoter methylation and gene expression of TCF21, which targets the TET family members and increases genome-wide 5hmC levels, thus inhibiting HCC progression (Dong et al., 2021). More importantly, circMEMO1 and its downstream target miR-106b-5p can increase the sensitivity of HCC cells to sorafenib treatment. CircTRIM33-12, an independent risk factor for the overall survival (OS) and recurrence-free survival (RFS) of patients with HCC after surgery, upregulates TET1 expression by sponging oncogenic miR-191 (Zhang et al., 2019c), and it has shown that TET1 can suppress the Wnt signaling pathway and repress HCC cell proliferation and invasion (Fan J. et al., 2018; Zhang et al., 2019c). Interestingly, the expression of miR-191 is regulated by DNA methylation, adding another layer of complexity to the miR-191/Tet1 regulatory axis. Additionally, miR-191 can aggravate HCC progression by directly targeting TIMP metallopeptidase inhibitor 3 (TIMP3) (He et al., 2011). MiR-17-3p and miR-181b-5p may also enhance HCC progression through repressing TIMP3, a well-known tumor suppressor of the oncogenic STAT3 signaling pathway (Shen et al., 2016; Yu et al., 2018; Zou et al., 2020; Tian et al., 2022). Interestingly, there is a concomitant reduction of TIMP3 and cSMARCA5 expression in HCC cells (Yu et al., 2018). Further mechanistic investigations revealed that cSMARCA5 can inhibit the TIMP3-STAT3 pathway by sponging miR-17-3p/miR-181b-5p, thereby suppressing the growth and metastasis of HCC (Shen et al., 2016; Yu et al., 2018). Circ-102,166, a circRNA encoded by the TEX2 gene locus, inhibited HCC development by binding with miR-182 and miR-184 to regulate the expression of their downstream targets, including FOXO3a, MTSS1, SOX7, p-RB and c-MYC (Li R. et al., 2020). CircLIFR has also been identified to function as a tumor suppressor, suppressing the proliferation and invasion of HCC cells through the miR-624-5p/GSK-3β axis (Yang L. et al., 2022).

Due to the considerable bioenergy, biosynthesis and redox requirements caused by rapid proliferation, cancer cells need to reprogram pathways of nutrient acquisition and metabolism. According to the Warburg effect, HCC cells converts aerobic oxidation to glycolysis through metabolic reprogramming, thereby supporting rapid proliferation (Liberti and Locasale, 2016). PI3K/AKT/mTOR-related circRNAs exert their essential functions in this process. As a downstream target of the PI3K-Akt-mTOR signaling pathway, pyruvate kinase (PK) participates in the final rate-limiting step of glycolysis by catalyzing phosphoenolpyruvate (PEP) and ADP to pyruvate and ATP. Especially, PKM2, one of PK isoforms, is overexpressed in malignant cells and enhances tumor progression through modulating metabolic reprogramming and other processes (Wei et al., 2016). The miR-338-8p degrades PKM2 mRNA by targeting its 3’-UTR. Li et al. demonstrated that circMAT2B acts as a miR-338-3p sponge to upregulate the expression level of PKM2, thus promoting glycolysis and glycolysis-related cell proliferation of HCC (Li Q. et al., 2019). CircRPN2 also regulates aerobic glycolysis reprogramming in HCC cells (Li J. et al., 2022). Enolase 1 (ENO1) promotes AKT activation to exert its metabolic effects (Dai et al., 2018). CircRPN2 decreases the level of phosphorylated AKT and mTOR by accelerating ubiquitin/proteasome-dependent ENO1 degradation, thus inhibiting HCC glycolysis and progression. On the other hand, circRPN2 suppresses HCC glycolysis, glucose uptake, ATP levels, and lactate production *via* the miR-183-5p/FOXO1 axis.

Moreover, several circRNAs can also modulate cell cycle and apoptosis to regulate tumor cell growth and proliferation. Cyclins and CDKs are two classes of regulators for cell cycle progression and always exert functions by binding to each other. Therefore, CDK inhibitors, such as P21 and P27, can regulate the cell cycle by inhibiting the function of CDKs (Levkau et al., 1998). For example, circ-CSPP1 was upregulated in HCC and increased the expression of cyclin E2 (CCNE2), which regulates the G1-to-S phase transition *via* the CCNE2/CDK2 axis (Kanska et al., 2016; Sun Q. et al., 2020). Hsa_circ_0000517 downregulation induced cell cycle arrest in HCC cells and decreased the expression of P21 and cyclin D1 (He S. et al., 2020). P21 can bind CDK2, thus regulating the cell cycle progression of G1 to S phase (Brugarolas et al., 1999). As reported by Du et al., circ-Foxo3 forms a circ-Foxo3-p21-CDK2 ternary complex with p21 and CDK2, which hijacks CDK2 together with p21, and inhibits the cyclin E/CDK2 complex formation, thus arresting the process from G1 to S phase (Du et al., 2016). At the same time, the ternary complex can also block the progression of the cell cycle in S phase by abolishing the inhibitory effect of p21 on the cyclin A/CDK2 complex (Du et al., 2016). Similar to the p21, p27 is known to play a key role in regulating CDK2 activity (Sherr and Roberts, 1995). Liu et al. demonstrated that circBACH1 represses p27 mRNA translation by gathering HuR, and consequently accelerates the entrance of HCC cells from G1 to S phase (Liu B. et al., 2020). Undoubtedly, cell proliferation is closely related to senescence, and circRNAs may be involved in this regulation. It has been proposed that circLARP4 could sponge miR761 and upregulate RUNX3 expression, thus activating the downstream p53/p21 signaling and arresting the G1/S cell cycle of HCC cells (Chen et al., 2019b). Not only cell cycle, but apoptosis is also regulated by p53, p21 and RUNX3 (Hotta et al., 1998; Dotto, 2000; Vousden and Lu, 2002). Therefore, it is reasonable to speculate that the effect of circRNAs on apoptosis is associated with HCC development. Indeed, circ-LRIG3 has been demonstrated to reduce apoptosis by forming a ternary complex with EZH2 and STAT3 (Sun et al., 2020b). Hsa_circRNA_104,348 directly targeted miR-187-3p, and thus suppressed apoptosis of HCC cells (Huang G. et al., 2020). These studies collectively suggest that the majority of circRNAs may function as miRNA sponges, which subsequently regulate downstream genes and signaling pathways associated with proliferation, senescence, and apoptosis of HCC cells.

### 4.3 CircRNAs regulate HCC invasion, migration and metastasis

Cancer-related mortality is predominantly due to the metastasis of primary tumor and subsequent recrudescence at the distinct site. CircRNAs can regulate tumor cell invasion and metastasis through multiple signaling pathways, such as MAPK (including extracellular regulated kinase) (Wang et al., 2018; Hu et al., 2020), TGF-β (Meng et al., 2018; Song et al., 2020), and AKT (Huang et al., 2020c). Protein phosphatase, Mg2+/Mn2+ dependent 1 F (PPM1F), a member of the PP2C family of Ser/Thr protein phosphatases, plays a vital role in HCC progression. PPM1F facilitates cell motility and invasiveness by inactivating p53 signaling and promoting tumor metastasis via the MAPK signaling and exosomal cytokine secretion (Susila et al., 2010; Zhang et al., 2017). CircSLC3A2 upregulates PPM1F expression by sponging miR-490-3p, activating the MAPK signaling pathway in HCC (Wang et al., 2018). Hu et al. determined that circASAP1 promotes HCC cell proliferation and metastasis by sponging miR-326 and miR-532-5p to enhance the expression of colony-stimulating factor-1 (CSF-1) and MAPK1, which are direct targets of miR-326 and miR-532-5p (Hu et al., 2020). The extracellular regulated kinase (ERK) 1/2 signaling pathway is highly involved in tumor growth and metastasis, and MAPK1 acts as a tumor promoter that exerts its function through the ERK1/2 pathway (Han et al., 2021). Circ_0001175 is increased in HCC tumors and facilitates HCC cell proliferation, migration, invasion, epithelial-mesenchymal transition (EMT) and lung metastasis through enhancing the expression of SNX5, a target of miR-130a-5p, resulted in activation of the EGFR-ERK1/2 pathway (Li L. et al., 2020). It has been fairly elucidated that EMT functions as a prominent step in the transition of early-stage HCC to aggressive and metastatic malignancies, increasing cancer cell motility (Brabletz et al., 2018). It has shown that elevated circMET expression induces EMT by modulating associated signaling pathways, such as the chemokine pathway, TNF pathway, and PI3K-Akt pathway (Huang et al., 2020c). CD44v6 and FOSL2 is EMT-stimulated genes that trigger the TGF-β signaling pathway and promote EMT. Circ0003998 can act as a ceRNA of miRNA-143-3p to enhance FOSL2 expression, while circ0003998 could bind with PCBP1-poly (rC) binding protein 1 (PCBP1) to increase the expression level of CD44v6 (Tripathi et al., 2016; Sun et al., 2018; Song et al., 2020), exemplifying the coordination of two circRNAs in HCC EMT regulation.

The EMT process is controlled by many protein-coding factors, such as ZIC1 and Twist, which may be modulated by ncRNAs including circRNAs. For example, circMTO1 can competitively bind to miR-541-50 and upregulate the downstream target ZIC1 expression, thereby suppressing HCC EMT (Li D. et al., 2021). ZIC1 is a zinc-finger transcription factor and has a major impact on the Wnt/β-catenin and EMT signaling networks. Briefly, ZIC1 attenuates the activation of Wnt/β-catenin signaling, consequently decreases the expression of mesenchymal marker proteins, including MMP2, N-cadherin and Vimentin. Vimentin, a significant constituent of intermediate filament proteins, functions as a potent enhancer of cell migration in cancerous tissues and facilitates EMT process (Ridge et al., 2022). As one of the EMT-inducing transcription factors, Twist 1 regulates the transcription of EMT-associated genes, including Vimentin. As reported by Meng et al. (Meng et al., 2018), Twist1 upregulates the level of Cul2 circular RNA (circ-10720), which sponges miRNAs targeting Vimentin and promotes EMT in HCC. Intriguingly, circ-10720-overexpressing cells is enriched in gene sets associated with VEGF and TGF-β receptor signaling pathway, suggesting a broad role of this circRNA in HCC EMT.

CircRNAs may also modulate HCC metastasis through regulating certain oncogenes and anti-oncogenes. CircPTTG1IP blocked miR-16-5p and subsequently upregulated its target RNF125, leading to ubiquitination and degradation of JAK1, and eventually reduced HCC invasion and metastasis (Peng et al., 2022). CircZNF566 promoted HCC tumorigenesis and metastasis by sponging miR-4738-3p to increase its target TDO2, an essential enzyme of Try metabolism, albeit the exact mechanisms remains unclear (Opitz et al., 2011; Li S. et al., 2020). The histone methyltransferase EZH2 is a key metastasis-related gene that can physically bind to a large amount of ncRNAs. Sun et al. found that circ-ADD3 inhibits HCC cell migration and invasion, as well as lung metastasis, by potentiating CDK1-mediated ubiquitination and degradation of EZH2 (Benetatos et al., 2013). Subsequently, the decrease of EZH2 significantly increases the expression of anti-metastatic genes, including circ-ADD3, thereby forming a regulatory loop to inhibit the metastasis of HCC (Sun et al., 2019). In addition, circRNAs also exert function by binding with RBPs directly. For example, circDLC1 interacts with the RNA-binding protein HuR and impairs its binding to matrix metallopeptidases 1 (MMP1) mRNAs, blocking the translation of MMP1 (Liu H. et al., 2021), a known repressor of HCC cell proliferation and invasion (Kim et al., 2018).

Angiogenesis can sustain the growing malignant tissues to obtain nutrients and oxygen and remove metabolic wastes continuously. Undoubtedly, the tumor-associated neovascularization system plays critical roles in tumor dissemination and metastasis. Increasing evidence has revealed that circRNAs can act as metastasis regulators by managing angiogenesis (Zhao et al., 2022). Interestingly, it has been demonstrated that circRNAs possess the ability to promote angiogenesis under the special condition, hypoxia, a typical characteristic of the tumor microenvironment (Boeckel et al., 2015). SRY (sex determining region Y)-box 9 (SOX9), can interact with β-catenin to inhibit the activation of Wnt/β-catenin signaling pathway which is involved in vasculogenic mimicry. Yang et al. discovered that cZNF292 interacts with SOX9 protein and blocks its nuclear translocation, which subsequently stimulates the Wnt/β-catenin signaling pathway activity, thereby promoting angiogenesis in human hepatoma SMMC7721 cells under hypoxia (Yang W. et al., 2019). CircGFRA1/miR-149 also plays a vital role in the angiogenesis of HCC, probaly through activating multiple miR-149 targets, such as PPM1F, AKT1, and LRIG2 (Zhang Y. et al., 2014; Ji et al., 2019; Yu Y. et al., 2020). Therefore, targeting circRNAs to disrupt the angiogenesis of HCC may be a hopeful therapeutic strategy.

## 5 CircRNA as a modulator of HCC microenvironment

The tumor microenvironment (TME) is essential in the pathobiology of cancer. The interaction between malignant and surrounding cells creates the TME, which plays a crucial role in all stages of cancer progression, metastasis and treatment.

### 5.1 CircRNAs regulate tumor immune microenvironment

**FIGURE 4:**
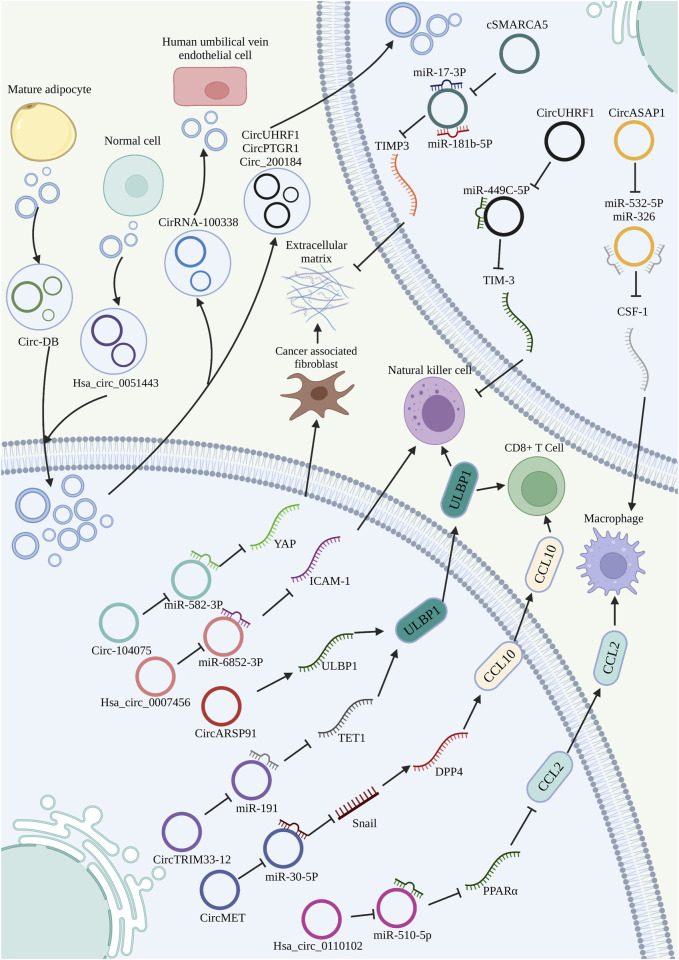
Role of circRNAs in the TME of HCC. CircRNAs promote or inhibit immune cell activity, and participate in ECM remodeling by regulating fibroblast recruitment and activation. Exosome-encapsulated circRNAs as modulators in intercellular communication of HCC.

Immune cells are the most abundant cellular components in TME and function as a double-edged sword in cancer-protecting the host and promoting tumor proliferation. Previous studies have shown that foreign circRNAs potently stimulate immune signaling and inhibit RNA virus infection (Chen et al., 2017) (Figure 4). Tumor-infiltrating lymphocytes (TILs) are an important part of the immune system and are positively correlated with patient’s survival, tumor size and metastasis (Weng et al., 2019). Through performing global circRNA microarray screening and qRT-PCR validations, Weng et al. found that hsa_circ_0064428 was significantly reduced in HCC patients with high TILs and negatively correlated with overall survival and metastasis (Weng et al., 2019). Among the plethora of lymphocytes, natural killer (NK) cells and cytotoxic CD8^+^ T (CTLs) cells are deemed as the ‘professional killers’ because of their involvement in the direct killing of pathogens (Trinchieri, 1989). Interestingly, NK cells can directly kill the target cells without prior exposure to antigen. Moreover, NK cells are considered the first line of defense for host immune surveillance and play a vital role in tumor immune microenvironment. In cellular stress conditions, active ligands on tumor cell surface are upregulated and interact with activating receptors on NK cells. In the end, both the NK cell-mediated cytotoxicity and their secreted pro-inflammatory cytokines cause the death of the target cells (Chan et al., 2014). Recent studies elucidated that circRNAs may participate in HCC immunity in this way. For example, circARSP91 upregulates the expression of UL16 binding protein 1 (ULBP1). Notably, ULBP1 serves as a killer cell lectin to facilitate NK cells to recognize and attack HCC cells. At the same time, hsa_circ_0007456 increases the susceptibility of HCC cells to NK cell cytotoxicity (Shi et al., 2021), indicating a vital role in tumor immunity. CTLs differentiate into effector cytotoxic CTLs and bind to cancer cells through the interaction between their T cell receptors and the specific neo-antigen-MHC class I complex, then killing the malignant cells by the Fas/FasL/caspase cascade pathway or directly secreting effector cytokines and cytotoxic granules (Halle et al., 2017). In a pan-cancer analysis, Zou et al. discovered that CDR1as might play a specific role in immune and stromal cell infiltration in tumor tissues, especially those of CD8+T cells and activated NK cells (Zou et al., 2019). Cancer cells secret chemokines or cytokines, such as CCL2 and CCL5, thus recruiting macrophages to the TME and polarizing to a tumor-associated macrophage (TAM) phenotype (Christofides et al., 2022). CircASAP1 promotes TAM infiltration by sponging miR-326 and miR-532-5p, and that CSF-1 is a common target of those two miRNAs (Hu et al., 2020). Hsa_circ_0110,102, was significantly downregulated in HCC, and inhibited macrophage activation and HCC progression. C-C chemokine ligand 2 (CCL2) was overexpressed in HCC by the activation of PPARα and interacted with C-C motif chemokine receptor 2 to regulate the chemotaxis of TAMs, contributing to cancer progression in HCC TME. Hsa_circ_0110,102 inhibits the expression and release of CCL2 into the microenvironment through the miR-580-5p/PPARα pathway, thereby alleviating macrophage recruitment and TAM (Wang X. et al., 2021). Furthermore, through spearman correlation analysis, Zhou et al. found that at least six circRNAs are related to macrophage infiltration in HCC, including hsa_circ_0091570, hsa_circ_0072088, hsa_circ_0004913, hsa_circ_0002980, hsa_circ_0001955 and hsa_circ_0000520 (Zhou et al., 2020).

### 5.2 CircRNAs regulate CAFs and ECM

Cancer-associated fibroblasts (CAFs) are involved in TME construction and promote cancer proliferation and metastasis. Interestingly, CDR1as was found to participate in regulating CAFs and extracellular matrix (ECM). Zou et al. identified that CDR1as expression is positively correlated with the infiltrating level of CAFs (Zou et al., 2019). This idea is backed up by another study (Du et al., 2016). Compare with cancer cell lines, the level of circ-Foxo3 was significantly reduced in fibroblast cells, such as NIH3T3 and mouse embryo fibroblasts (Du et al., 2016). In HCC tissues, HNF4a, a well-acknowledged HCC-promoting transcription factor, stimulates the expression of circ_104,075 by binding with its promoter. In addition, circ_104,075 can act as a ceRNA to upregulate YAP expression by absorbing miR-582-3p, which may cause the remodel of ECM by inducing CAFs, and promote the proliferation of HCC (Zhang et al., 2018d). ECM, consisting of collagens, proteoglycans, elastin, fibronectin, laminins, and several other glycoproteins, is one of the significant components of TME and continuously undergoes remodeling. cSMARCA5, whose expression is reduced in HCC, prevents degradation of the ECM of HCC by inhibiting the activity of MMPs (Yu et al., 2018). Several circRNAs are reported to be involved in ECM remodeling in other cancers, and it is of interest to explore their roles in HCC TME.

### 5.3 Exosomal circRNAs in HCC microenvironment

Exosomes are small bioactive vesicles produced by endocytosis in the cell membrane, and serve as a nanoscale messenger shuttling between cells to transfer information and cargos. The peculiarity that exosomes are secreted into surrounding body fluids make them play a vital role in various pathophysiological changes. CircRNA-sequencing on exosomes derived from liver cancer cells showed that circRNAs were enriched in exosomes compared with normal cells. Meanwhile, the enrichment degree of tumor circRNAs in serum was even related to tumor size (Li Y. et al., 2015). This phenomenon suggests that exosomal circRNAs are likely involved in the proliferation and metastasis of HCC (Figure 4). Several circRNAs are secreted into the TME by HCC cells. CircUHRF1, an exosomal circRNA secreted by HCC cells, can sponge miR-449c-5p to promote the expression of TIM-3, a receptor on NK cell surface, resulting in the loss of NK cell activity (Vivier et al., 2011). Thus, exosomal cireUHRF1 inhibits NK cell activity and facilitates HCC progression (Zhang P. F. et al., 2020). Huang et al. found that exosomal circRNA-100338 enhances HCC cell proliferation, invasion and angiogenesis *via* the mTOR signaling pathway. The HCC-derived exosomal circRNA-100338 can affect the proliferation of human umbilical vein endothelial cells (Huang et al., 2020c). Exosomal circPTGR1 from HCC-LM3 cells enhances cell migration and invasion through the miR449a-MET pathway. Acting as messengers to enhance intercellular communication is an important function of exosomes. Interestingly, exosomal circRNAs derived from normal tissues may be also involved in HCC progression. For example, exosomal circRNA hsa_circ_0051443, originated from normal cells, suppresses the progression of HCC *via* the miR-331-3p/BAK1 axis (Chen L. et al., 2020). Ubiquitin-specific protease 7 (USP7), a deubiquitinating enzyme, is associated with the proliferation and invasion of cancers, including HCC (Novellasdemunt et al., 2017). Circ-DB, an exosomal circRNA derived from the adipose tissue, can promote the growth of HCC by regulating the miR-34a/USP7 axis and deubiquitination (Zhang H. et al., 2019). Of note, exosomes are broadly existed in surrounding body fluids, as mentioned above, suggesting that exosoaml cirRNAs may be used as potential biomarkers for cancerdiagnosis.

## 6 CircRNAs in HCC-related diseases

Chronic infection with hepatitis viruses is one of the main risk factors for HCC, especially hepatitis B virus (HBV) and hepatitis C virus (HCV). HBV infection is the most prominent risk factor for HCC development, accounting for ∼50% of cases (Akinyemiju et al., 2017). Interestingly, a novel circRNA HBV_circ_1 produced by HBV pgRNA/pcRNA, rather than host mRNAs, was identified in HBV-positive HepG2 cells and HBV-related HCC tissues (Zhu et al., 2021). HBV_circ_1 upregulates CDK1 expression by directly binding to CDK1 *in vitro* and *in vivo*, and CDK1 then binds to cyclins and activates cell cycle progression. These results suggest that the interaction of HBV_circ_1 with CDK1 may promote HCC development, although the specific mechanism remains further studied. As to HCV, unique to this virus is a dependence on the liver-specific miR-122. The binding of miR-122 to two binding sites in the 5’ UTR of HCV RNA is critical for viral replication by moderately stimulating viral protein translation (Henke et al., 2008) and protecting the uncapped HCV RNA genome from exoribonuclease Xrn2 degradation (Sedano and Sarnow, 2014). This provides a new approach for HCV treatment by competitively inhibiting miR-122 binding to HCV, thus restraining its replication and translation. Based on the property of circRNAs as miRNA sponges, Jots et al. produced artificial circRNA sponges *in vitro* that efficiently sequester miR-122, thereby inhibiting viral protein in an HCV cell culture system (Jost et al., 2018). The stability of circRNAs is a precious advantage in scientific research and clinical application.

Non-alcoholic fatty liver disease (NAFLD) is the leading etiology of non-cirrhotic HCC (Ioannou, 2021). NAFLD is emerging as the most common cause of chronic liver disease worldwide. It is a multifaceted disorder that ranges from the simple accumulation of triglycerides in hepatocytes (hepatic steatosis) to steatosis with inflammation, non-alcoholic steatohepatitis (NASH), fibrosis and cirrhosis, which may evolve towards HCC. Many molecules play important roles in both NAFLD/NASH and HCC, including ncRNAs (Fu et al., 2014; Xu et al., 2021; Tian et al., 2022). Emerging evidence illustrates that oxidative stress caused by impaired mitochondrial function is involved in the pathogenesis of NAFLD, including NASH (Sunny et al., 2017). Mitochondrial dysfunction leads to an excessive production of reactive oxygen species (ROS), which is released from mitochondria into the cytoplasm and activates human liver fibroblasts, thus promoting the fibrosis process of NAFLD/NASH (Zhao Q. et al., 2020). Interestingly, circRNAs are the crucial mediators in inhibiting ROS leakage from mitochondria and blocking fibroblast activation. The circRNA, steatohepatitis-associated circRNA ATP synthase subunit b (ATP5B) regulator (SCAR), originates from the circularization of the second exon of its host gene (JA760602) and localizes in the mitochondria matrix. SCAR directly binds to ATP5B, a regulator of mitochondrial permeability transition pore (mPTP), leading to the closure of mPTP. mPTP is an indispensable channel for mitochondrial ROS to enter the cytoplasm. Accordingly, SCAR can modulate mitochondrial function by controlling the release of mROS and the activation of liver fibroblasts to regulate the progression of steatosis to NASH (Zhao Q. et al., 2020). It is well known that PPARα and associated signaling pathways are involved in the pathogenesis of NAFLD, and the expression level of PPARα is inversely proportional to the severity of NASH (Francque et al., 2015; Huang K. et al., 2017). CircRNA_0046366, circRNA_0046367 and PPARα shared the complementary sequences with miR-34a. Further studies found that circRNA_0046366 and circRNA_0046367 can serve as the endogenous sponges of miR-34a and then activate PPARα. Activation of hepatic PPARα attenuated lipid peroxidation and mitochondrial injury by promoting the transcriptional activation of multiple genes for lipid metabolism, such as CPT1A, SLC27A, CPT2 and ACBD3 (Guo et al., 2017a; Guo et al., 2018). Currently, several circRNAs have been identified in HCC-related diseases, but their relationship to disease transformation remains unclear (Table 2).

**TABLE 2:** Dysregulated circRNAs in HCC related disease.

Disease	CircRNAs	Target/Pathway	Expression change	PMID	Reference
Viral hepatitis	HBV_circ_1	HBV_circ_1/CDK1	Upregulated	34,589,285	Zhu et al. (2021)
	hsa_circ_0000650	hsa_circ_0000650/miR-6873-3p/TGF-β2	Downregulated	29,888,838	Zhou et al. (2018b)
	circPSD3	circPSD3/eIF4A3/nonsense-mediated decay (NMD) pathway	Upregulated	32,764,824	Chen et al. (2020b)
	circRNA-100338	circRNA-100338/miR-141-3p/MTSS1	Upregulated	28,710,406	Huang et al. (2017b)
	hsa_circ_0004812	hsa_circ_0004812/miR-1287-5p/FSTL1	Upregulated	32,188,476	Zhang and Wang (2020)
	circ_0027089	circ_0027089/miR-136-5p/NACC1	Upregulated	34,419,960	He et al. (2022)
	circ_RNF13 (hsa_circ_0067717)	circ_RNF13/miR-424-5p/TGIF2	Upregulated	33,714,261	Chen et al. (2021b)
NAFLD	circRNA SCAR	circRNA SCAR/ATP5B/mPTP	Downregulated	32,931,733	Zhao et al. (2020b)
	circRNA_0046366	circRNA_0046366/miR-34a/PPARα	Downregulated	29,391,755	Guo et al. (2018)
	circRNA_0046367	circRNA_0046367/miR-34a/PPARα	Downregulated	29,018,509	Guo et al. (2017a)
	circScd1	JAK2/STAT5	Downregulated	30,259,280	Li et al. (2019a)
	circRNA_0049392	circRNA_0049392/miR-7037-5p, miR-6919-5p	Upregulated	33,308,253	Yuan et al. (2020)
	circRNA_021412	circRNA_021412/miR-1972/LPIN1	Downregulated	28,717,649	Guo et al. (2017b)
Hepatic fibrosis	ccirc-PWWP2A	ccirc-PWWP2A/miR-203, miR-223/Fstl1, TLR4	Upregulated	31,719,209	Liu et al. (2019f)
	circRNA_0067835	CircRNA_0067835/miR-155/FoxO3	Upregulated	30,481,761	Zhu et al. (2018)
	circFBXW7	circFBXW7/miR-18b-3p/FBXW7	Downregulated	32,308,754	Chen et al. (2020e)
	hsa_circ_0070963	hsa_circ_0070963/miR-223-3p/LEMD3	Downregulated	32,003,753	Ji et al. (2020)
	circMTO1	circMTO1/miR-17-5p/Smad7	Downregulated	31,148,365	Wang et al. (2019c)
	circPSD3	circPSD3/miR-92b-3p/Smad7	Downregulated	33,614,234	Bu et al. (2021)

## 7 CircRNAs as diagnostic and prognositic biomarkers for HCC

**FIGURE 5:**
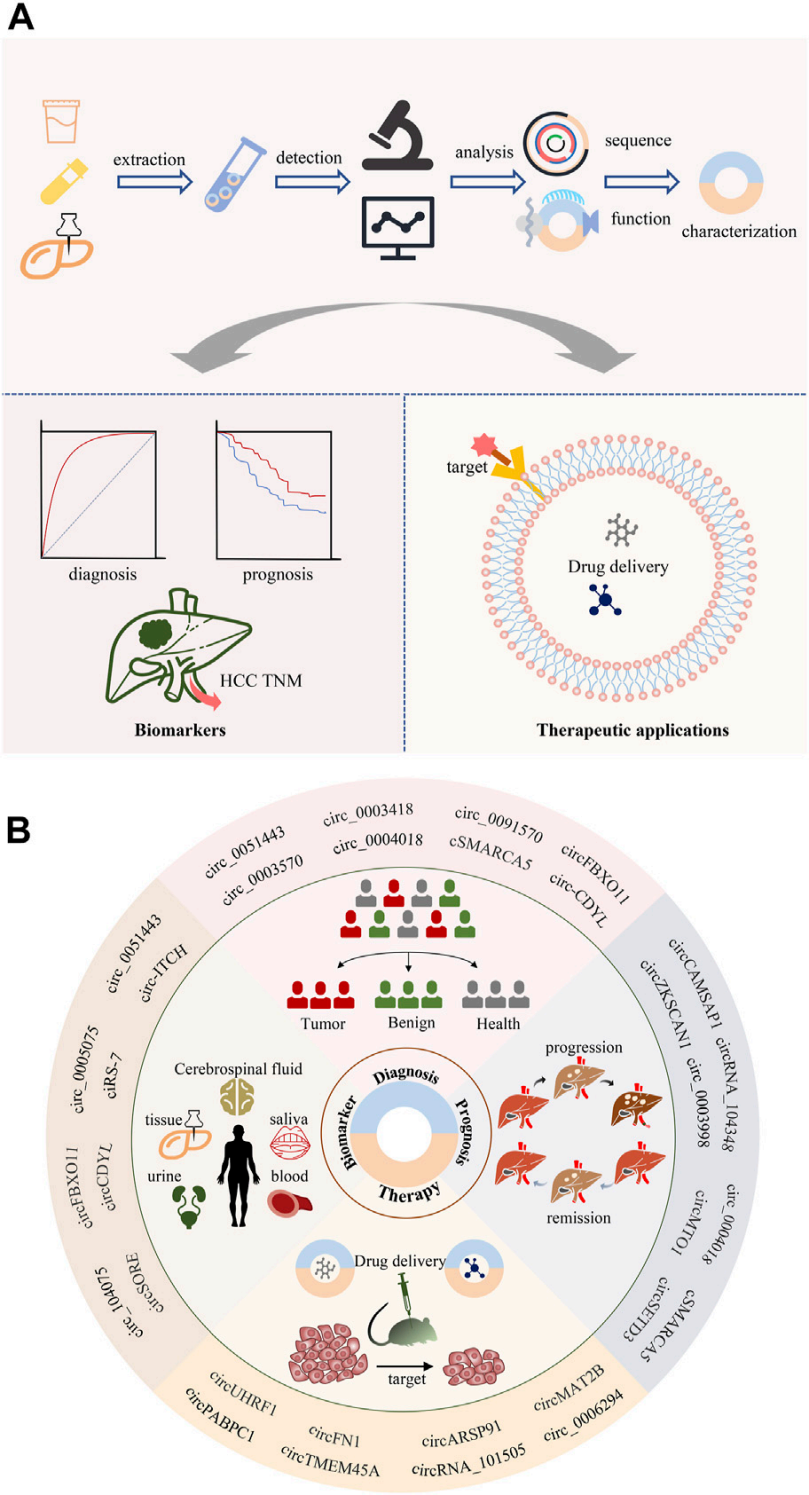
Clinical application of circRNAs. **(A)** CircRNA biomarkers can be isolated from blood, as well as from in the body fluids and tissues. **(B)** CircRNAs may serve as potential biomarkers for diagnosis, prognosis, and therapy selection of cancer.

Diagnosis at early stages is crucial for successful curative treatment. Still, the early diagnosis of HCC remains a challenge due to the scarcity of symptoms until progression to late-stage. Meanwhile, the prognosis for HCC remains dismal because of its aggressiveness and high recurrence rate. Therefore, effective biomarkers are required to improve early diagnosis and prognosis analysis. CircRNAs are shielded from exonuclease destruction because of their circular form, and thus have a substantially longer half-life than parent mRNAs—about 48 h as opposed to 10 h (Kristensen et al., 2019). Additionally, circRNAs exhibit cell- and tissue-specific expression patterns and are extraordinarily stable and preserved. Importantly, circRNAs have been found in bodily tissues and fluids, rais the possibility that they might be used as prognostic and diagnostic biomarkers at the molecular levels (Figure 5).

Serum alpha-fetoprotein (AFP) is still the most common traditional serum biomarker for HCC screening and diagnosis. With the research on circRNAs, its application may be challenged. It has been shown that the area under the ROC curve (AUC-ROC: 0.973), a sensitivity (SE) of 96.0% and a specificity (SP) of 98.3% indicated that circ_104,075 might be a better serum biomarker for the diagnosis for HCC compared to the AFP (AUC-ROC: 0.750, SE69.3%, SP68.3%) (Zhang et al., 2018d). Furthermore, the use of AFP alone is constrained since AFP may be high in cirrhosis or hepatitis and not all HCC cells release AFP (Wang and Zhang, 2020). In comparison to the single standard biomarker, combinations of circRNAs and AFP may show greater diagnostic or prognostic accuracy. For example, the plasma level of hsa_circ_0003998 in HCC patients was significantly higher than in hepatitis B patients and healthy controls. Its level was significantly reduced after the operation, with an AUC value of 0.892 alone and 0.947 in combination with AFP for discriminating between HCC and healthy controls.

Recent studies have conducted that exosomes may serve as promising candidates for liquid biopsy, particularly for monitoring and forecasting tumor occurrence and metastasis (Kalluri and LeBleu, 2020). Exosomal circRNAs may be a unique and promising type of biomarkers for the early detection of cancer since they are numerous and stable in exosomes (Wang S. et al., 2021). For example, circ_0051443 has been found to have a significantly lower expression level in plasma exosomes from patients than in those from healthy controls. This biomarker can be used for both prediction and diagnosis (Chen W. et al., 2020).

Significantly, with the development of high-throughput sequencing techniques and public genetic databases, many circRNA-based prognostic models for HCC have been established. Zhang et al. employed The Cancer Genome Atlas (TCGA) and Gene Expression Omnibus (GEO), and constructed a prognostic signature with seven target mRNAs by univariate, lasso and multivariate Cox regression analyses. According to the targeting relationship between 7 hub mRNAs and other RNAs, the circRNA-lncRNA-miRNA-mRNA ceRNA prognostic network was constructed, comprising of 21 circRNAs (Zhang et al., 2021b). Meanwhile, Huang et al. obtained an alternative prognostic model of HCC. Differentially expressed circRNAs and mRNAs in normal liver tissues and HCC were constructed into the ceRNA network, Significant hub nodes in the ceRNA network were 5 circRNAs, including hsa_circ_0004662, hsa_circ_0005735, hsa_circ_0006990, hsa_circ_0018403 and hsa_circ_0100,609. In addition, according to the mRNA expression level difference in this ceRNA network, a prognostic risk assessment tool, based on the expressions of 7 genes (PLOD2, TARS, RNF19B, CCT2, RAN, C5orf30 and MCM10), has been developed (Han L. et al., 2022). To sum up, the ceRNA network is the main form of prognostic model of HCC, which has a promising clinical application.

In total, the above examples demonstrated that circRNAs could be considered as promising diagnostic and prognostic biomarkers in different body fluids for HCC patients. However, the research of circRNAs as cancer biomarkers is still in its infancy, and some challenges remains on their clinical application. Confirmation of a marker as an indicator requires retrospective and prospective analyses of a large-scale sample to make statistics. In addition, the normalization of circRNAs requires repeated tests to determine the cut-off value and reference range. Because of the substantial ongoing improvements, we suppose that most of these queries will be overcome and that more circRNAs will be detected and applied in the clinic in the future. Table 3 list some circRNAs in application in HCC.

**TABLE 3:** Dysregulated circRNAs in HCC act as novel potential diagnostic and/or prognostic biomarkers.

CircRNA	Sample	Expression	Clinical application	Clinical relevance	PMID	Refrence
circRNA_104075	Serum	Up-regulated	Diagnostic	AUC = 0.973 Sensitivity = 96.0% Specificity = 98.3%	30361504	Zhang et al. (2018d)
hsa_circTRIM11_001 (hsa_circ_0016788)	Tissue	Up-regulated	Diagnostic	AUC = 0.851	29923236	Guan et al. (2018)
circSLC3A2 (hsa_circ_0022587)	Tissue	Up-regulated	Prognostic	High expression with poor OS (*p* = 0.0385)	30470261	Wang et al. (2018)
circ_0000798	PBMCs, peripheral blood mononuclear cells	Up-regulated	Diagnostic Prognostic	AUC = 0.703 High expression with poor OS (*p* < 0.0001)	30714679	Lei et al. (2019)
circADAMTS13	Tissue	Down-regulated	Prognostic	AUC = 0.987 Low expression with poor RFS (*p* = 0.044)	30537115	Qiu et al. (2019)
hsa_circ_0001649	Tissue	Down-regulated	Diagnostic Prognostic	AUC = 0.63 Sensitivity = 81% Specificity = 69% Low expression with poor OS (*p* = 0.015)	26600397	Qin et al. (2016)
circHIAT1	Tissue	Down-regulated	Prognostic	Low expression with poor OS (*p* = 0.003)	31108351	Wang et al. (2019e)
circSETD3 (hsa_circ_0000567)	Tissue	Down-regulated	Diagnostic Prognostic	AUC = 0.637 Low expression with poor OS (*p* = 0.013)	30795787	Xu et al. (2019)
circMAT2B	Tissue	Up-regulated	Prognostic	High expression with poor OS (*p* = 0.0011)	31004447	Li et al. (2019b)
hsa_circ_0078602	Tissue	Down-regulated	Diagnostic Prognostic	AUC = 0.787 Low expression with poor OS (*p* = 0.035)	30675276	Kou et al. (2019)
circRNA_104718	Tissue	Up-regulated	Prognostic	High expression with poor OS (*p* = 0.0245)	31278132	Yu et al. (2019)
circ_0000517	Tissue	Up-regulated	Prognostic	High expression with poor OS (*p* = 0.0069)	32774154	He et al. (2020a)
circ-BIRC6	Tissue	Up-regulated	Prognostic	High expression with poor OS (*p* < 0.05)	30931701	Yang et al. (2019b)
hsa-circ_0008450	Tissue	Up-regulated	Prognosis	High expression with poor OS (*p* = 0.0034)	30556306	Zhang et al. (2019b)
circRHOT1 (hsa_circRNA_102034)	Tissue	Up-regulated	Prognostic	High expression with poor OS (*p* = 0.002) and poor RFS (*p* = 0.002)	31324186	Wang et al. (2019b)
circRNA101505	Tissue	Down-regulated	Prognostic	Low expression with poor OS (*p* = 0.0005)	31372241	Luo et al. (2019a)
circASAP1 (hsa_circ_0085616)	Tissue	Up-regulated	Prognostic	High expression with poor OS (*p* = 0.001)	31838741	Luo et al. (2019a)
circ-ADD3	Plasma	Down-regulated	Diagnostic Prognostic	AUC = 0.8878 Low expression with poor OS (*p* = 0.0063) and poor RFS (*p* = 0.0084)	31497351	Sun et al. (2019)
circ-LRIG3 (hsa_circ_0027345)	Plasma	Up-regulated	Diagnostic Prognostic	AUC = 0.8681 high expression with poor OS (*p* = 0.0026) and poor RSF (*p* = 0.009)	33222697	Sun et al. (2020b)
circ-0051443	Exosome	Down-regulated	Diagnostic	AUC = 0.8089	32014458	Chen et al. (2020b)
hsa_circRNA8662-12 (circTRIM33-12)	Tissue	Down-regulated	Prognostic	Low expression with poor OS (*p* = 0.0007) and poor RFS (*p* = 0.0015)	31153371	Zhang et al. (2019c)
circLARP4	Tissue	Down-regulated	Prognostic	Low expression with poor OS (*p* = 0.001) and poor RFS (*p* = 0.004)	30520539	Chen et al. (2019c)
hsa_circ_0001727 (circZKSCAN1)	Tissue	Down-regulated	Diagnostic	AUC = 0.834 Sensitivity = 82.2% Specificity = 72.4%	28211215	Yao et al. (2017)
		Down-regulated	Prognostic	Low expression with poor OS (*p* < 0.001) and poor RFS (*p* < 0.001)	31281495	Zhu et al. (2019b)
hsa_circ_0003998	Tissue, Serum	Up-regulated	Diagnostic Prognostic	AUC = 0.894 Sensitivity = 84% Specificity = 80% (T) +AFP (AUC = 0.947; Se = 0.88; Sp = 0.92) (S) High expression with poor OS *p* = 0.005 (T)	31410028	Qiao et al. (2019)
circ-FOXP1	Serum	Up-regulated	Diagnostic	AUC = 0.9318 High expression with poor OS (*p* = 0.005)	31698267	Wang et al. (2020)
hsa_circ_0000976 hsa_circ_0007750 hsa_circ_0139897	Plasma	Up-regulated	Diagnostic	AUC = 0.843 Sensitivity = 87.5% Specificity = 81.2% AFP (AUC = 0.747; Se = 60.5%; Sp = 89.0%) +AFP (AUC = 0.863; Se = 92.8%; Sp = 79.9%)	31456215	Yu et al. (2020a)
hsa_circ_0028502	Tissue	Down-regulated	Diagnostic	AUC = 0.675 Sensitivity = 72.1% Specificity = 58.0%	31595711	Jiang et al. (2019)
hsa_circ_0076251	Tissue	Down-regulated	Diagnostic Prognostic	AUC = 0.738 Sensitivity = 71.3% Specificity = 64.0% Low expression with poor OS (*p* < 0.05)	31595711	Jiang et al. (2019)
hsa_circ_0027089	Plasma	Up-regulated	Diagnostic	AUC = 0.784 Sensitivity = 57.8% Specificity = 84.8% AFP (AUC = 0.857; Se = 68.75; Sp = 95.5%) +AFP (AUC = 0.80; Se = 79.69%; Sp = 82.14%)	31535687	Zhu et al. (2020b)
circ_AKT3	Exosome	Up-regulated	Prognostic	High expression with poor OS (*p* = 0.00014) and poor RFS (*p* = 0.0035)	32561093	Luo et al. (2020)
hsa_circ_0000267	Tissue	Up-regulated	Prognostic	High expression with poor OS (*p* = 0.001)	30719761	Pan et al. (2019)
cSMARCA5 (hsa_circ_0001445)	Tissue	Down-regulated	Prognostic	Low expression with poor OS (*p* = *0.0004)* and poor RFS (*p* = 0008)	29378234	Yu et al. (2018)
hsa_circ_0003570	Tissue	Down-regulated	Prognostic	Low expression with poor OS (*p* = 0.017) and poor PFS (*p* = 0.048)	36011395	Jang et al. (2022)
circGPR137B	Tissue	Down-regulated	Prognostic	Low expression with poor OS (*p* = 0.008) and early stage ones (*p* = 0.001)	35858900	Liu et al. (2022d)
circ_0000437	Tissue, Serum	Up-regulated	Diagnostic Prognostic	AUC = 0.9281 High expression with poor OS (*p* = 0.0379)	35730467	Chen et al. (2022a)
circ_LIFR	Tissue	Down-regulated	Prognostic	Low expression with poor OS (p = 0.0022) and poor RFS (*p* = 0.0035)	35581180	Yang et al. (2022a)

## 8 CircRNAs as therapeutic agents or targets

Advances in surgery and chemotherapy have improved the prognosis of patients with early-stage HCC (Peng et al., 2021). The majority of patients are diagnosed at the advanced stages of the disease and thus have to undergo chemotherapy (Vasan et al., 2019). The important emerging role of circRNAs in the initiation and progression of HCC makes them an attractive therapeutic target option. Several strategies based on the functions of circRNAs have been proposed to treat HCC. For instance, circARSP91 could function as a progression inhibitor to prevent androgen receptor (AR)-induced HCC cell migration and invasion. Targeting the AR/ADAR1/circARSP91 axis could be a potential new route for the development of therapies for HCC (Shi et al., 2017). Meanwhile, several intracellular expression systems, such as the Tornado which transforms the RNA into a loop by modifying its two ends, have been developed to efficiently increase circRNA expression in cells (Litke and Jaffrey, 2019). Another effective and convenient strategy, synthetic (artificial) circRNAs, also has potential therapeutic application in human patients. As one of the main functions of circRNAs is to sponge miRNAs, synthetic circRNAs containing miRNA binding sites were proved to achieve targeted loss of miRNA function and suppress cancer development (Loan Young et al., 2023). Significantly, synthetic circRNAs display excellent capability in terms of both quantity of protein produced and stability of production (Wesselhoeft et al., 2018). In addition, modified exosomes might be an effective approach safely delivering synthetic circRNAs *in vivo* because (Li C. et al., 2021).

Additionally, a number of techniques may be used to target circRNAs that are favorably linked to carcinogenesis or HCC development in order to influence therapy or enhance prognosis. To downregulate the production of circRNA, siRNA or shRNA usually target the particular backspliced sequence. Similarly, to counteract the effects of circRNAs, antisense oligonucleotides and the CRISPR/Cas9 system might be utilized (Piwecka et al., 2017). A recent study employed DNAzymes, which had circRNA-cleaving properties, to disable circRNAs sequences implicated in HCC and inhibit its cancer promoting ability (Ding et al., 2019).

Although HCC is one of the most frequent cancers, there have been very few drugs available that improve survival, and the efficiency of HCC chemotherapy is usually limited by intrinsic and acquired resistance. Sorafenib, one of the most widely used drugs for patients with non-resectable tumors, inhibits tumor cell proliferation and angiogenesis and promotes tumor cell apoptosis by targeting multiple receptor tyrosine kinases (RTKs), representing an effective first-line therapy for HCC (Llovet et al., 2022). However, the development of its resistance greatly minimizes its therapeutic benefits (Schlacher, 2018). Therefore, elucidating the molecular mechanism of drug resistance is important for cancer treatment. Given the broad involvement of miRNAs in cancer chemotherapy resistance, there is no doubt that circRNAs play a variety of roles in HCC drug resistance. For instance, circFN1, which is overexpressed in HCC cells, contributes to sorafenib resistance by regulating the miR-1205/E2F1 signaling pathway, which may be targeted to improve sensitivity to the multi-kinase inhibitor (Yang et al., 2020). Thus, circRNAs may be applied as targets for drug sensitization since a rising number of drug-resistant circRNAs associated with HCC have been found (Table 4).

**TABLE 4:** A list of dysregulated circRNAs related to drug resistance in HCC.

CircRNAs	Expression	Role in HCC	Target/Pathway	Effect on drug resistance	PMID	References
circFN1 (hsa_circ_0058124)	Upregulated	Oncogene	miR-1205/E2F1	Resistance to sorafenib	33,230,446	Yang et al. (2020)
hsa_circ_0006294	Downregulated	--	--	Resistance to sorafenib	32,226,514	Wu et al. (2020a)
hsa_circ_0035944						
circRNA-SORE	Upregulated	Oncogene	YBX1	Resistance to sorafenib	33,361,760	Xu et al. (2020a)
			miR-103a-2-5p and miR-660-3p/Wnt/β-catenin pathway	Resistance to sorafenib	33,222,692	Xu et al. (2020b)
circ003418	Downregulated	Suppressor	Wnt/β-catenin pathway	Sensitivity to cisplatin	31,807,029	Chen et al. (2019a)
hsa_circRNA_102,049	Downregulated	Suppressor	miR-214-3p/RELN	Sensitivity to sorafenib	35,034,536	Wang et al. (2022c)
circMEMO1	Downregulated	Suppressor	miR-106b-5p/TCF21/TET	Sensitivity to sorafenib	33,985,545	Dong et al. (2021)
circ0031242	Upregulated	Oncogene	miR-924/POU3F2	Resistance to cisplatin	33,531,841	Fan et al. (2021)
circARNT2	Upregulated	Oncogene	miR-155-5p/PDK1	Sensitivity to cisplatin	33,425,483	Li et al. (2021c)
circRNA102272	Upregulated	Oncogene	miR-326/RUNX2221	Resistance to cisplatin	33,324,096	Guan et al. (2020)
hsa_circ_0088364	Upregulated	Oncogene	--	Sensitivity to nitidine chloride	31,506,425	Xiong et al. (2019)
hsa_circ_0090049						
circFBXO11	Upregulated	Oncogene	miR-605/FOXO3/ABCB1	Resistance to Oxaliplatin (OXA)	32,222,024	Li et al. (2020a)
circSOD2	Upregulated	Oncogene	miR-497-5p/ANXA11	Resistance to PD-1	36,008,700	Ye et al. (2022)
hsa_circ_G004213	Downregulated	Suppressor	miR-513b-5p/PRPF39	Sensitivity to cisplatin	33,864,660	Qin et al. (2021)
circFOXM1	Downregulated	Suppressor	miR-1324/MECP2	Sensitivity to sorafenib	33,614,231	Weng et al. (2021)

## 9 Conclusion and perspectives

Nearly two-thirds of individuals with HCC have advanced stages of the disease when they are first identified, making HCC one of the most prevalent malignancies and the second leading cause of cancer mortality globally (Llovet et al., 2021c). Thousands of highly conserved endogenous circRNAs have been found in mammalian cells, despite previously being assumed to be a “mistake” in RNA splicing. Numerous research has demonstrated the extent to which circRNAs is systematically changed in cancer, and the significance of circRNAs in different cellular and biological processes, as well as tumorigenesis and metastasis, in HCC has been thoroughly documented. In this review, we briefly summarized the biological functions of circRNAs and their implications in HCC. The broad modification of circRNAs will open up a plethora of new avenues for diagnosis, therapy, and prognosis, all of which are critical issues of HCC care.

However, there are still many questions that need to be clarified. Firstly, at present, the nomenclature of circRNAs has not been unified. The chances are that the same molecule may be under research simultaneously while in different names, especially when multiple databases have been developed for circRNA analysis in this field (Table 5). Through these databases, researchers can search basic information about circRNAs, predict interactions of circRNAs with target molecules and their translation potential, and evaluate their relationships with diseases. Therefore, it is urgent to establish the uniformed name rule of circRNAs. Secondly, the studies discussed above have confirmed that circRNAs display altered expression patterns in HCC, their cell-type origins are not yet fully explored. This is very challenging because HCC is a complicated condition, especially the TME and the limitation of probe design targeting the back-spliced junction. Even though the effects of circRNAs on the TME have been extensively studied, it remains unclear how TME might affect the machinery of circRNAs biogenesis, secretion, transfer, and mode of action. Therefore, discriminating the cellular origins of secreted circRNAs to identify those involved in tissue function in health and dysfunction in disease; identifying potential circRNAs mediating inter-cell communications as well as those that may impact homeostasis in case of organ failure, and characterizing natural circRNAs carriers to reach distant organs may be part of the solution to these hurdles. Thirdly, the potential role of exosomal circRNAs in the development of HCC remains largely unknown even some investigations have shown their potential correlations. Exosomal circRNAs have been suggested as circulating biomarkers for cancer diagnosis as they have been shown to discriminate patients with cancer from healthy controls as shown above (Chen W. et al., 2020; Liu J. et al., 2022). However, effectively extracting circRNAs with low abundance from exosomes is a challenge, for instance how many copies of circRNA molecules in exosomes need to be evaluated. While concerning circRNAs found in adipose tissues, they may also be released to the circulation inside macrovesicles and have functions in target organs. Therefore, more *in vitro* and *in vivo* modeling of exosome-mediated circRNA communications, along with development of sensitive bioinformatic methods and mathematical mass-action models to capture all circRNA–target interactions, will undoubtedly provide insight into the function of these exosomal RNA species. Fourthly, functional peptides produced by circRNAs have emerged into scientific attention. As described above, β-catenin-370aa, encoded by circβ-catenin, activates the Wnt/β-catenin pathway and promotes liver cancer growth (Liang et al., 2019). Evidence has certificated the existence and function of circRNA-encoded peptides. However, high-throughput analytical and systematic detection methods such as ribosome profiling have technical challenges to such peptides. The similarity between circRNA-encoded peptides and the counterpart of mRNA makes them hard to distinguish. The identification of small peptides requires specific biochemical and bioinformatics methods seldom applied in genome-wide characterization. Moreover, cell- and tissue-specific expression complicate these assays. Thus, further stoichiometric analysis is required to explore these peptides’ functions better. Notably, it is also of interest to determine if circRNAs could be translated in the nucleus (Zou et al., 2023b). Fifth, it is speculated that the aberrant expression of circRNAs observed in cancer might be affected by epigenetic changes. Many circRNAs have protein-coding potential, and the translation process can be driven by m^6^A (Yang Y. et al., 2017). Meanwhile, m^6^A can affect the function of circRNA via changing the methylation state of downstream molecules. As one of the most abundant RNA modifications, m^6^A provides us with an intermediate mechanism by which circRNAs are regulated by upstream molecules and allows us to predict and interfere with disease progression caused by the dysregulation of circRNAs. There is no doubt that it would greatly expand our understanding on circRNAs and accelerate their applications. Further, up to date, no specific biological functions have been detected in the majority of already discovered circRNAs, which is also one of the reasons that circRNAs were regarded as by-products of splicing when first discovered (Nigro et al., 1991). Considering the ubiquitous m^6^A modification in annotated functional circRNAs, it is possible that the role of circRNA might be related to their tissue and developmental stage specificity. Several circRNAs displaying differential expression in specific tissues, developmental stages and subcellular locations may attribute to certain epigenetic and environmental mechanisms (Wu et al., 2019; Tang et al., 2023). In addition, recent evidence suggests an important role of certain RNA secondary structures, such as RNA G-quadruplex, in gene expression (Liu G. et al., 2020; Varshney et al., 2020; Liu G. et al., 2022; Tong et al., 2023). However, the implications of these regulatory modes in circRNA expression await further investigation. Sixthly, it is too soon to speculate on the viability of these molecules as biomarkers and therapeutic disease targets in a clinical HCC scenario. CircRNAs are being investigated for their potential value in the diagnosis, prognosis, and treatments of HCC. The creation of these methods is mostly based on the expression levels of particular circRNAs, although it is not known whether certain biomarkers are unique to HCC or prevalent in other malignancies. The efficacy of circRNAs has not been investigated in sizable, clinically monitored, and definitive cohorts, nor have their mechanisms of action been thoroughly investigated. Additionally, there are still questions regarding possible toxicity, pharmacological reaction, immunologic rejection, accumulation in other tissues, and sustained long-term effects. But studies have shown that exosomes could significantly broaden the uses of circRNAs (Dai et al., 2020). Nevertheless, a better understanding of these issues is required to see circRNAs clinical translation becoming a reality. Last but not least, given the complexity of cancer pathomechanisms, relying on the pattern of a single circRNA paradigm to diagnose and treat HCC may not be biologically maximally relevant and specific enough to provide clinical utility, even studies combining circRNAs with traditional cancer biomarkers for diagnosis have been conducted as mentioned above. Rather, one should consider a multi-markers approach that combines candidate circRNAs/signatures, miRNAs, RBPs, traditional cancer biomarkers, which may be highly discriminative, accurate, and efficient in predicting HCC.

**TABLE 5:** Summarization of the circRNAs related databases.

Databases	Description	Link or access	Country/Region	PMID	Reference
NPInter v3.0	Supplies experimentally verified functional interactions among ncRNAs (except tRNAs and rRNAs) and other biomolecules (proteins, RNAs and DNAs)	http://www.bioinfo.org/NPInter/Database	China	27,087,310	Hao et al. (2016)
CircBase	Provides datasets and scripts to identify known and novel circRNAs in sequence-based data	http://www.circbase.org	Germany	36,230,902	Glažar et al. (2014)
CSCD	Collects RNASeq datasets in cancer, normal and commom samples and provides an integrated circRNA database to benefit functional studies of cancer-specific circRNAs	http://gb.whu.edu.cn/CSCD	China	29,036,403	Xia et al. (2018)
CircNet	Provides novel circRNAs, integrated miRNA-target networks, expression profiles of circRNA isoforms, genomic annotations of circRNA isoforms and sequences of circRNA isoforms	http://circnet.mbc.nctu.edu.tw	China	26,450,965	Liu et al. (2016)
Circ2Disease	A manually curated database of experimentally validated circRNAs in human disease	http://bioinformatics.zju.edu.cn/Circ2Disease/circRNAgroup.html	China	30,030,469	Yao et al. (2018)
CircR2Disease	Provides circRNA dysregulation in disease and circRNA-miRNA regulation netmork	http://bioinfo.snnu.edu.cn/CircR2Disease	China	29,741,596	Fan et al. (2018a)
CCRDB	A cancer circRNAs-related database and its application in hepatocellular carcinoma-related circRNAs	nc.ude.usys.liam@hxdssi	China	31,219,565	Liu et al. (2019e)
Circ2Traits	Provides the potential association of circRNAs with human traits and trait-related SNPs on circRNA loci	http://gyanxet-beta.com/circdb/	China	24,339,831	Ghosal et al. (2013)
CircRNADb	A comprehensive database for human circular RNAs with protein-coding annotations	http://reprod.njmu.edu.cn/circrnadb	China	27,725,737	Chen et al. (2016)
DeepBase v2.0	Identification, expression, evolution and function of small RNAs, lncRNAs and circRNAs from deep-sequencing data	http://biocenter.sysu.edu.cn/deepBase/	China	19,966,272	Yang et al. (2010)
TSCD	Provides a global view of tissue-specific circRNAs in main tissues of human and mouse that is able to contribute to identify new markers for organogenesis, development, and disease	http://gb.whu.edu.cn/TSCD	China	27,543,790	Xia et al. (2017)
StarBase V2.0	Decoding miRNA-ceRNA, miRNA-ncRNA and protein-RNA interaction networks from large-scale CLIP-Seq data	http://starbase.sysu.edu.cn/	China	24,297,251	Li et al. (2014)
CIRCpedia v2	An updated comprehensive database containing circRNA annotations from over 180 RNA-seq datasets across six different species	http://www.picb.ac.cn/rnomics/circpedia/	China	30,172,046	(Dong et al., 2018)
ExoRBase	A repository of circRNA, lncRNA and mRNA derived from RNA-seq data analyses of human blood exosomes	http://www.exoRBase.org	China	30,053,265	Li et al. (2018b)
HumanViCe	Provides the potential ceRNA networks in virus infected human cells	http://gyanxet-beta.com/humanvice	India	25,120,561	Ghosal et al. (2014)
CirclncRNAnet	Maps functional networks of long or circular forms of ncRNAs and supports the uploading and processing of user-defined NGS-based gene expression matrix data	http://app.cgu.edu.tw/circlnc	China	29,194,536	Wu et al. (2018)
CircInteractome	Explores potential interactions of circRNAs with RBPs; designs specific divergent primers to detect circRNAs; studies tissue- and cell-specific circRNAs; identify gene-specific circRNAs; explores potential miRNAs interacting with circRNAs, and designs specific siRNAs to silence circRNAs	https://circinteractome.nia.nih.gov/	United States	29,322,439	Panda et al. (2018)
TRCirc	Provides other regulatory information about transcription of circRNAs, including their expression, methylation levels, H3K27ac signals in regulatory regions and super-enhancers associated with circRNAs	http://www.licpathway.net/TRCirc	China	30,184,150	Tang et al. (2019)
The Landscape of Circular RNA Expression in the Human Brain	Provides a comprehensive catalogue of circRNAs as well as a deeper insight into their expression in the human brain, which are available as a free resource	http://www.voineagulab.unsw.edu.au/circ_rna	Australia	31,570,194	Gokool et al. (2020)
CircAtlas v2.0	Integrates expression and functional profiles in vertebrates from millions of circRNAs across 7 species (human, macaca, mouse, rat, pig, chicken, dog)	http://circatlas.biols.ac.cn	China	32,345,360	Wu et al. (2020b)
TransCirc	Integrates various direct and indirect evidences to predict coding potential of each human circRNA and the putative translation products	https://www.biosino.org/transcirc	China	33,074,314	Huang et al. (2021)
MiOncoCirc	The first extensive clinical, cancer-centric resource of circRNAs; largely constructed from clinical tumor samples (2,000+) across more than 40 cancer sites; queries mutations and copy-number; novel biomarkers can be nominated	https://mioncocirc.github.io/	United States	30,735,636	Vo et al. (2019)
Targetscan	Analyse circRNA-miRNA-mRNA networks for new circRNAs	http://www.targetscan.org/vert_72/	Germany	27,141,961	Kuleshov et al. (2016)
SomamiR 2.0	Provides information and functional analysis of expected miRNA– ceRNA interaction (miRNA-circRNA)	http://compbio.uthsc.edu/SomamiR	India	26,578,591	Bhattacharya and Cui (2016)
circRNA in fluids	Visualizes each circRNA in fluids	https://mulongdu.shinyapps.io/circrnas_in_fluids/	China	33,430,880	Wang et al. (2021b)
CircView	Detects circRNAs by existing tools and regulatory elements among 5 species (human, mouse, zebrafish, fly, and worm)	http://gb.whu.edu.cn/CircView/	China	33,141,028	Saaoud et al. (2021)
CircBank	Comprehensive description for human circRNAs function especially m6A modification and mutation of circRNAs	http://www.circbank.cn/	China	31,023,147	Liu et al. (2019d)

Taken all, circRNAs are a new type of ncRNAs that have a variety of activities. Experimental studies conducted *in vivo* and *in vitro* have shown evidence that circRNAs have a role in the control of HCC. To fully comprehend their importance, effectiveness, generalizability, security, and reliability, tremendous investigations are required. It is advisable to employ multiple tools for analysis because different tools have varying degrees of efficiency. Moreover, the improvement in the high-throughput RNA sequencing and the cost reduction, coupled with the establishment of an unbiased bioinformatics analysis, will lead to the identification of a large number of novel circRNAs, which will accelerate the clinical application of circRNAs in the comprehensive management of HCC.
